# Vitamin B12 levels in thyroid disorders: A systematic review and meta-analysis

**DOI:** 10.3389/fendo.2023.1070592

**Published:** 2023-02-22

**Authors:** Vicente A. Benites-Zapata, Felipe L. Ignacio-Cconchoy, Juan R. Ulloque-Badaracco, Enrique A. Hernandez-Bustamante, Esteban A. Alarcón-Braga, Ali Al-kassab-Córdova, Percy Herrera-Añazco

**Affiliations:** ^1^ Doctorado en Nutrición y Alimentos, Universidad San Ignacio de Loyola, Lima, Peru; ^2^ Unidad de Investigación para la Generacióny Síntesis de Evidencias en Salud, Vicerrectorado de Investigación, Universidad San Ignacio de Loyola, Lima, Peru; ^3^ Escuela de Medicina, Universidad Peruana de Ciencias Aplicadas, Lima, Peru; ^4^ Sociedad Científica de Estudiantes de Medicina de la Universidad Nacional de Trujillo, Trujillo, Peru; ^5^ Grupo Peruano de Investigación Epidemiológica, Unidad para la Generación y Síntesis de Evidencias en Salud, Universidad San Ignacio de Loyola, Lima, Peru; ^6^ Sociedad Científica de Estudiantes de Medicina de la Universidad Peruana de Ciencias Aplicadas, Lima, Peru; ^7^ Centro de Excelencia en Investigaciones Económicas y Sociales en Salud, Universidad San Ignacio de Loyola, Lima, Peru; ^8^ Universidad Privada del Norte, Trujillo, Peru

**Keywords:** thyroid, vitamin B12, autoimmune thyroid disease, hypothyroidism, hyperthyroidism, subclinical hypothyroidism

## Abstract

**Background and aims:**

Numerous studies have found an association between vitamin deficiency and thyroid disorders (TD). The presence of anti-parietal cell antibodies is indicative of reduced ability to absorb vitamin B12. Thus, this study reviewed the existing studies with the objective of assessing differences in the serum levels of vitamin B12 among patients with and without TD, the frequency of vitamin B12 deficiency in patients with TD, and the presence of anti-parietal cell antibodies in patients with TD.

**Methods:**

A meta-analysis of random-effects model was conducted to calculate pooled frequencies, mean differences (MD), and their respective 95% confidence intervals (CI). We identified 64 studies that met our inclusion criteria (n = 28597).

**Results:**

We found that patients with hypothyroidism had lower vitamin B12 levels than healthy participants (MD: −60.67 pg/mL; 95% CI: −107.31 to −14.03 pg/mL; p = 0.01). No significant differences in vitamin B12 levels were observed between healthy participants and patients with hyperthyroidism (p = 0.78), autoimmune thyroid disease (AITD) (p = 0.22), or subclinical hypothyroidism (SH) (p = 0.79). The frequencies of vitamin B12 deficiency among patients with hypothyroidism, hyperthyroidism, SH, and AITD were 27%, 6%, 27%, and 18%, respectively.

**Conclusions:**

Patients with hypothyroidism had lower levels of vitamin B12 than healthy participants. No significant differences were observed between vitamin B12 levels and hyperthyroidism, AITD, or SH.

**Systematic Review Registration:**

https://www.crd.york.ac.uk/prospero/display_record.php?RecordID=324422, identifier (CRD42022324422).

## 1 Introduction

Thyroid disorders (TD) are a heterogeneous group of diseases that affect the thyroid’s anatomy or function (1), including hypothyroidism, hyperthyroidism, subclinical hypothyroidism (SH), subclinical hyperthyroidism, structural abnormalities, and cancer (1, 2). The increasing life expectancy of the global population has significantly increased the incidence of TD and its global burden, especially among older adults (3). The frequency and incidence of TD differ among regions. However, it has been estimated that some TD, such as hypothyroidism, affect 5% of the global population (4), whereas hyperthyroidism affects 0.8% and 1.3% of the population in Europe and the USA, respectively (2, 5). Also, the global age-standardized thyroid cancer (TC) rates are 10.1 per 100 000 women and 3.1 per 100 000 men (6).

Thyroid function is regulated by various nutrients, primarily iodine and selenium. Iodine is an essential micronutrient required for thyroid hormone synthesis, whereas selenium is a cofactor of thyroid enzymes (7, 8). Certain vitamins also play moderating roles in thyroid function, such as vitamins A, E, D and B. Previous studies have reported vitamin deficiencies in patients with TD (7, 9). Regarding the B complex vitamins, B12 is one of the most important as it is indispensable to several biochemical processes. In fact, Vitamin B12, or cobalamin, plays a central role in hematopoiesis and is a component of enzymes, such as methylmalonyl-coenzyme. Although the causes of vitamin B12 deficiency in patients with TD may be multifactorial, they would be predominantly related to the comorbidity of other autoimmune disorders and dietary habits (10–12).

The intrinsic factor of Castle is a mucoprotein essential for the absorption of vitamin B12 at the distal ileum that is synthesized and secreted by the parietal cells of the stomach (13). Therefore, these cells play a key role in pathologies associated with vitamin B12 deficiency, such as pernicious anemia (PA) and autoimmune atrophic gastritis (AAG). Thus, the detection of anti-parietal cell antibodies (APCA) has emerged as a means for screening these pathologies (14).

As aforementioned, the frequency of TD has been increasing in the last decades. Thus, an in-depth assessment of the vitamin B12 serum levels among patients with and without TD, frequency of vitamin B12 deficiency, and presence of APCA in patients with TD has great clinical relevance and impact. Even though a narrative review described the association between vitamin B12 levels and TD (15), there was not an adequate data search and selection strategy, which are necessary to systematize the available evidence on this association. Therefore, the main objective of this systematic review was to evaluate the differences in the serum levels of vitamin B12 among patients with and without TD. The secondary objectives were to evaluate the frequency of vitamin B12 deficiency in patients with TD and the frequency of APCA in patients with autoimmune thyroid diseases (AITD).

## 2 Methods

### 2.1 Registration and search strategy

This systematic review was conducted in accordance with the tenets of the Preferred Reporting Items for Systematic Reviews and Meta-Analyses (PRISMA) statement (16) and the Cochrane Handbook for Systematic Reviews. In addition, a summary of the protocol was registered with the International Prospective Register of Systematic Reviews (PROSPERO) [CRD42022324422].

A systematic search was performed in four databases (PubMed, Scopus, Web of Science, and Embase) on April 3, 2022, with no restrictions regarding language or year of publication. The search included the following keywords: “thyroid diseases” and “vitamin B12”. We also conducted a manual search on preprint platforms (medRxiv and Research Square) and other databases (CINAHL, China National Knowledge Internet databases, Wanfang Database, and Scielo).

### 2.2 Eligibility criteria

We included studies on adult participants (≥18 years) that met the following criteria: (1) studies assessing the frequency of B12 deficiency in patients with TD, (2) studies evaluating differences in the B12 levels between patients with TD and healthy participants, and (3) studies evaluating the frequency of APCA in patients with TD. We excluded: (1) case reports, (2) editorials, and (3) any type of review.

### 2.3 Study selection

The articles obtained from the electronic search were uploaded to the data management software Rayyan QCRI (Rayyan Systems Inc. ^©^, Cambridge, MA, USA). Four of the authors (VAB-Z, JRU-B, EA-B, and EAH-B) independently screened the titles and abstracts of each article to identify potentially eligible studies. Then, they read the full text of the articles identified in the previous stage to find those that met our selection criteria. All studies that did not fully meet the selection criteria were excluded from our review. Any disagreements were resolved by discussion until reaching a consensus among all authors.

### 2.4 Data extraction

A standardized data collection sheet was created in Microsoft Excel. Two authors (AA-C and PH-A) independently extracted the following information from each article: title, author, year, country, number of participants, age, sex, vitamin B12 assay method, vitamin B12 levels (pg/mL) of healthy participants, vitamin B12 levels of patients with TD, frequency of vitamin B12 deficiency, and frequency of APCA (+). In cases of missing information, the corresponding author was contacted *via* email to request the missing data.

### 2.5 Quality assessment

The quality of each study was independently assessed by four reviewers (VAB-Z, JRUB, AA-C, and EAH-B) using the Newcastle–Ottawa Scale (NOS) for the cohort and case–control studies, and an adaptation of the NOS for cross-sectional studies (NOS-CS) (17, 18). Both scales consist of a checklist covering three domains: selection, comparability, and outcome/exposure. For this study, articles with a score of seven or more on these scales were deemed to have a low risk of bias, whereas those with a score of less than seven were deemed to have a high risk of bias. This is the rating system recommended for the NOS and NOS-CS. In case of disagreements over the rating of a study, all authors examined the article and reached a consensus.

### 2.6 Statistical analysis

The information obtained from the included articles was combined using the Review Manager v.5.4 (The Cochrane Collaboration, Copenhagen, Denmark) and STATA v.17.0 software (College Station, TX: StataCorp LLC). All the meta-analyses were conducted using a random-effects model. The DerSimonian and Laird method was employed to estimate the between-study variance. For the pooled analysis of mean differences (MD), the data from those studies that used medians and interquartile ranges (IQR) were converted to means and standard deviations (SD) using Hozo’s method (19). For variables with the standard errors (SE) reported, SD was determined using the following equation: SE × √ (sample size). For the pooled analysis of proportions, we employed the Clopper–Pearson method to calculate the 95% confidence intervals (CI) and the Freeman–Tukey double arcsine transformation as the variance-stabilizing transformation. The between-study heterogeneity was evaluated using a chi-squared test and the I^2^ statistic. For the chi-squared test, P-values < 0.1 were considered indicative of heterogeneity. For the I^2^ statistic, heterogeneity was classified as low if I^2^ < 30%, moderate if I^2^ = 30%–60%, and high if I^2^ > 60%. We conducted subgroup analyses based on the continents where the studies were carried out. Also, sensitivity analysis was also conducted eliminating studies with a high risk of bias. Finally, publication bias was assessed through funnel plots and Egger’s test.

## 3 Results

### 3.1 Search results

**Figure 1:**
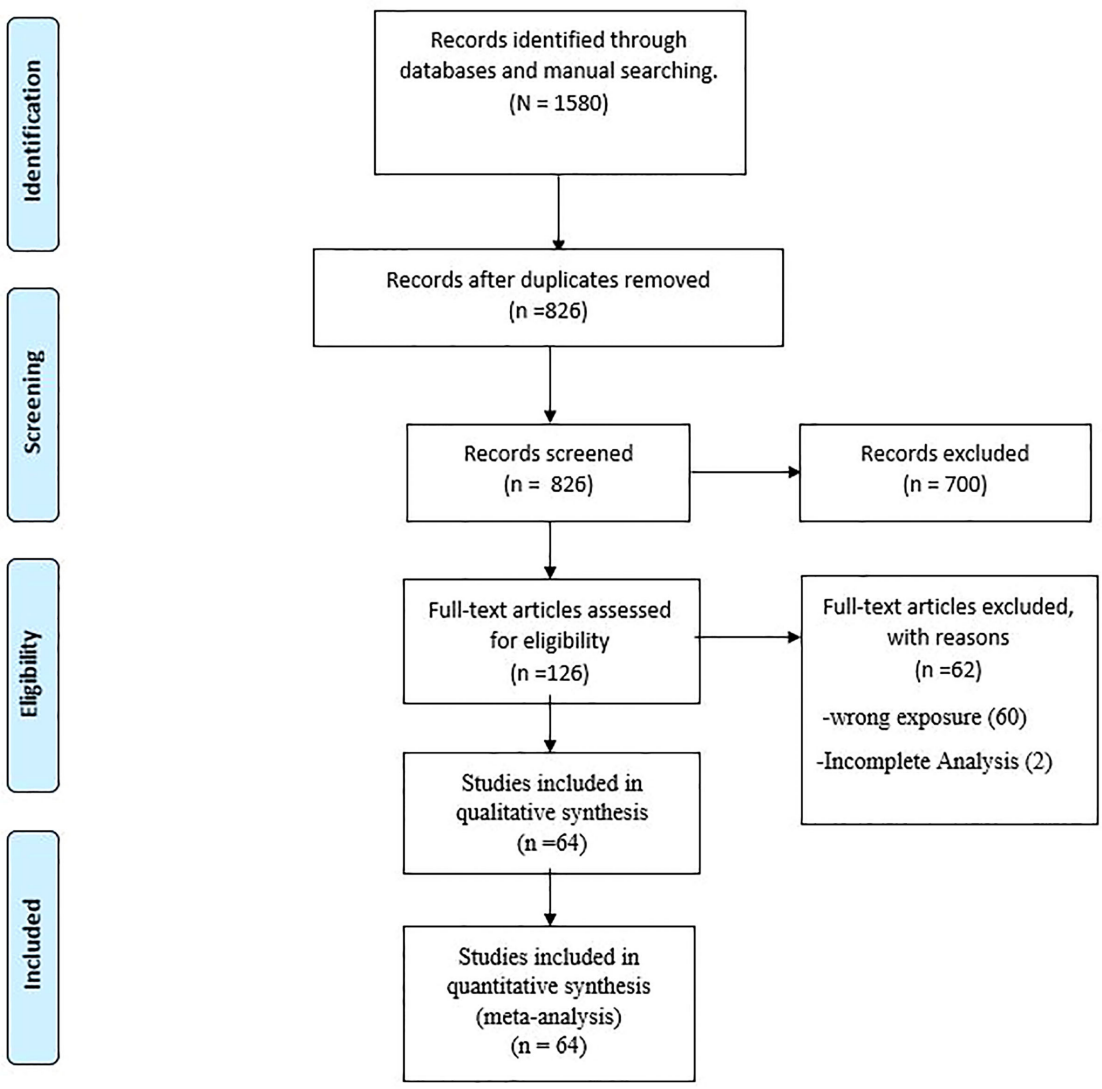
PRISMA Flow Diagram.

Our electronic search identified 1580 articles, from which 754 duplicates were excluded. The screening of the titles and abstracts led to the exclusion of a further 700 studies. The whole manuscript assessments resulted in the exclusion of 62 studies. Finally, a total of 64 studies were included in our systematic review and meta-analysis (12, 20–82). A flowchart of the selection process is presented in **Figure 1**.

### 3.2 Study characteristics

The characteristics of the included studies are summarized in **Table 1**. We included 64 studies published between 1967 and 2022 in 20 countries. A total of 28597 participants (14915 male and 13682 female) were evaluated; however, 12 studies did not report the number of participants by gender. The participants were aged 19–94 years; yet, 15 studies did not provide this information. As well, the cut-off points for defining vitamin B12 deficiency were reported in 31 studies and ranged from 130 to 400 pg/mL.

**Table 1:** Characteristics of the studies included in this review.

Author	Year	Country	Participants (female/male)	Mean/median age (SD/IQR)	Threshold for vitamin B12 deficiency (pg/mL)	TD evaluated	Vitamin B12 levels in healthy participants Mean (SD)	Vitamin B12 levels in patients with TD Mean (SD)	Patients with TD and vitamin B12 deficiency (frequency %)	Patients with TD and normal/high levels of vitamin B12 (frequency %)	Vitamin B12 assay method	Patients with AITD and APCA (+) (frequency %)	Patients with AITD and APCA(-) (frequency %)
***Das et al.***	2012	India	60(42/18)	36(19 - 67)	NR	Hypothyroidism	NR	NR	9(15)	51(85)	NR	NR	NR
***Wang et al.***	2012	Taiwan	380(346/34)	62 (12)	<200	AITD	701.9(181.4)	665.5(286)	12(6.32)	178(93.68)	NR	48(12.7)	332(87.3)
***Velarde-Mayol et al.***	2014	Spain	409(342/34)	78(8)	<199	AITD	NR	NR	76(18.58)	333(81.42)	NR	NR	NR
***Venerito et al.***	2015	Germany	34(NR/NR)	55 (13)	NR	AITD	NR	NR	1(2.94)	33(97.06)	NR	11(32.35)	23(64.65)
***Bhuta et al.***	2019	India	60(48/12)	NR(NR)	<210	Hypothyroidism	NR	NR	17(28.3)	43(71.7)	FEI	NR	NR
***Sattar-Lakho et al.***	2018	Pakistan	145(48/97)	42(8.9)	<150	Hypothyroidism	NR	NR	105(72.41)	40(27.6)	NR	NR	NR
***Kumari et al.***	2015	India	350(250/100)	32 (11)	<200	AITD	NR	NR	194(55.43)	156 (44.57)	NR	NR	NR
***Carrol et al.***	2015	United States of America	80(61/19)	55(17)	<200	AITD	655.8 (342.7)	606.8 (281.8)	0. 0(0)	36 (100)	ECLIA	4(11.11)	32(88.89)
***Siddique et al.***	2017	Pakistan	225(122/103)	47(7)	NR	Hypothyroidism	NR	NR	54(24)	171(76)	CLIA	NR	NR
***Jabbar et al.***	2008	Pakistan	116(95/21)	44 (13)	<200	Hypothyroidism	NR	NR	47(41.52)	69(59.48)	RIA	NR	NR
***Jabeen et al.***	2016	Pakistan	204(197/7)	37 (11)	<200	Hypothyroidism	NR	NR	112(54.9)	92(45.1)	RIA	NR	NR
***Adnan et al.***	2019	Iraq	70(NR/NR)	NR(NR)	<400	Hypothyroidism	788.62(138.21)	462.06(224.93)	NR	NR	Spectrophotometry	NR	NR
***Şanver et al.***	2022	Turkey	261(261/0)	46(15)	NR	AITD	377(287)	351(188)	NR	NR	CLIA	NR	NR
***Twito et al.***	2015	Israel	120(108/12)	50(16)	NR	AITD	NR	NR	NR	NR	NR	34(28.3)	86(71.3)
***Utiyama et al.***	2017	Brazil	243(213/30)	45 (13)	NR	AITD	NR	NR	NR	NR	NR	49(20.16)	194(75.84)
***Howel et al.***	1967	England	74(65/9)	NR(NR)	NR	AITD	NR	NR	NR	NR	NR	5(6.76)	69(93.24)
***Tozzoli et al***	2010	Italy	208(187/21)	43(29)	NR	AITD	NR	NR	NR	NR	NR	51(24.5)	157(75.48)
***Gerenova et al.***	2013	Bulgaria	151(142/9)	49(1.2)	NR	AITD	NR	NR	NR	NR	NR	51(33.77)	100(66.23)
***Yadav et al.***	2019	India	100(88/12)	33(7)	<211	Hypothyroidism	NR	NR	12(12)	88(88)	NR	NR	NR
***Checchi et al.***	2008	Italy	391(351/40)	55.3(15)	NR	AITD	NR	NR	NR	NR	NR	155(39.6)	236(60.4)
***Lahner et al.***	2008	Italy	128(107/21)	54 (20–76)	NR	AITD	NR	NR	NR	NR	NR	110(86.7)	18(13.3)
***Chan et al.***	2009	China	56(40/16)	75(46)	NR	AITD	NR	NR	NR	NR	NR	37(66.07)	19(33.93)
***Morawiec-Szymonik et al.***	2019	Poland	51(35/16)	NR(NR)	NR	AITD	NR	NR	NR	NR	NR	29(56.86)	22(43.14)
***Khan et al.***	2019	India	75(45/30)	NR(NR)	<211	Hypothyroidism	NR	NR	45(60)	30(40)	NR	NR	NR
***Colleran et al.***	2003	United States of America	31(NR/NR)	NR(NR)	<200	Hyperthyroidism	418 (219)	514 (210)	1(4.76)	20(95.24)	CLIA	NR	NR
***Alperin et al.***	1970	United States of America	88(NR/NR)	NR(NR)	<200	Hyperthyroidism	572(183)	347(144)	2(5.9)	32(94.1)	NR	NR	NR
***Nitu et al.***	2016	India	100(100/0)	NR(NR)	NR	Hyperthyroidism	276.08 (120.01)	341.5(25.58 )	NR	NR	HPLC	NR	NR
***Kumar et al.***	2019	India	400(NR/NR)	NR(NR)	NR	Hypothyroidism	456.43(243.54)	211.34(121.45)	NR	NR	CLIA	NR	NR
***Tripathi et al.***	2019	India	350(214/136)	33.78(13.9)	NR	Hypothyroidism	483.93(264.7 4)	210.45(129.3)	NR	NR	NR	NR	NR
***Berker et al.***	2009	Turkey	42(42/0)	24 (2.8)	NR	Hyperthyroidism	235 (86.9)	209.5 (36.3)	0(0)	42(100)	CLIA	NR	NR
***Khubchandani et al.***	2015	India	100(72/28)	39.48(14.19)	<200	Hypothyroidism	365.17(45.82)	187.38(35.89)	32(64)	18(36)	CLIA	NR	NR
***Choudhary et al.***	2021	India	150(86/64)	NR(NR)	<200	Hypothyroidism	NR	NR	38(25.3)	112(74.7)	NR	NR	NR
***Garcia-Garcia***	2010	Spain	148(137/11)	45(15)	NR	AITD	NR	NR	NR	NR	NR	30(20.27)	118(79.73)
***Castoro et al.***	2016	Italy	242(207/35)	41 (12–78)	NR	AITD	NR	NR	NR	NR	NR	57(23.55)	185(76.45)
***Alexandraki et al.***	2014	Greece	120(98/22)	51 (13)	NR	AITD	NR	NR	NR	NR	NR	38(31.67)	82(68.33)
***Souka et al.***	2018	United Arab Emirates	60(60/0)	40.8(9.5)	NR	Hypothyroidism	371.75(201.48)	279.25(362.9)	NR	NR	ECLIA	NR	NR
***Ozmen et al.***	2006	Turkey	47(35/12)	51(35–66)	NR	Hypothyroidism	474.93(327.21)	475.61(172.63)	NR	NR	CLIA	NR	NR
***Min-Yu et al.***	2014	South Korea	17541(6209/11332)	41.8 (10)	NR	Hypothyroidism	421(95.3)	423 (91.3)	NR	NR	CLIA	NR	NR
						SH	421(95.3)	423 (91.3)	NR	NR			
***Srikrishna et al.***	2015	India	440(377/63)	48.41(11.65)	<211	Hypothyroidism	412(93)	327(90)	NR	NR	ECLIA	NR	NR
						SH	412(93)	393(99)	NR	NR			
***Sengul et al.***	2004	Turkey	58(58/0)	42.3 (10.85)	NR	Hypothyroidism	241.4 (33.47)	250.69 (110.13)	NR	NR	CLIA	NR	NR
						SH	241.4 (33.47)	250.69 (110.13)	NR	NR			
***Luboshitzky et al.***	2002	Israel	91(91/0)	48(13)	<192	Hypothyroidism	298(135)	317(140)	NR	NR	CLIA	NR	NR
						SH	298(135)	317(140)	NR	NR			
***Çakal et al.***	2007	Turkey	46(NR/NR)	41.4(14.1)	<145	Hypothyroidism	244.2(47.2)	236.4(102)	NR	NR	CLIA	NR	NR
						SH	244.2(47.2)	245.1(88.8)	NR	NR			
***Nedrebo et al.***	1998	Norway	438(250/188)	48(19–89)	NR	Hypothyroidism	508.13(39.02)	724.93(122.45)	NR	NR	CLIA	NR	NR
						Hyperthyroidism	508.13(39.02)	577.23(104.37)	NR	NR			
***Diekman et al.***	2001	Netherlands	96(75/21)	38 (22–79)	NR	Hypothyroidism	410.56(161.24)	453.92(253.38)	NR	NR	RIA	NR	NR
						Hyperthyroidism	471.54(197.83)	462.05(188.34)	NR	NR			
***Calcaterra et al.***	2019	Italy	220(184/36)	NR(NR)	NR	AITD	NR	NR	4(40)	6(60)	CLIA	10(4.55)	210(95.45)
						Hypothyroidism	NR	NR	3(37.5)	5(62.5)			
						Hyperthyroidism	NR	NR	1(33.3)	2(66.7)			
***Dagdelen et al.***	2012	Turkey	327(NR/NR)	NR(NR)	<200	AITD	NR	NR	51(15.6)	276(84.4)	NR	NR	NR
						Hypothyroidism	NR	NR	50(18.25)	224(81.75)			
						Hyperthyroidism	NR	NR	1(1.9)	52(98.1)			
***Leineweber et al.***	2016	United States of America	494(NR/NR)	NR(NR)	<200	AITD	NR	NR	19(4.8)	379(95.2)	NR	88(22.1)	310(77.9)
						Hypothyroidism	NR	NR	18(4)	439(96)			
						Hyperthyroidism	NR	NR	4(10.8)	33(89.2)			
***Meling et al.***	2022	Norway	458(331/127)	NR(NR)	<200	AITD	NR	NR	48(10.5)	410(89.5)	NR	NR	NR
						Hypothyroidism	NR	NR	36(9.5)	344(90.5)			
						Hyperthyroidism	NR	NR	12(15.4)	66(84.6)			
***Wiebolt et al.***	2011	Netherlands	882(751/132)	50(14)	<130	AITD	NR	NR	66(8.85)	680(91.15)	NR	73(12.2)	525(87.8)
						Hypothyroidism	NR	NR	30(8.8)	310(91.2)			
						Hyperthyroidism	NR	NR	36(7.6)	436(92.4)			
***Raju et al.***	2021	India	50(27/23)	45(12.8)	<200	AITD	NR	NR	17(70)	7(30)	RIA	NR	NR
						Hypothyroidism	NR	NR	26(52)	24(48)			
						SH	NR	NR	16(57.14)	12(42.86)			
***Ness-Abramof et al.***	2006	Israel	115(108/7)	47 (15)	<133	AITD	NR	NR	32(27.8)	83(72.2)	CLIA	NR	NR
						Hypothyroidism	NR	NR	27(28.7)	67(71.3)			
						Hyperthyroidism	NR	NR	3(17.6)	14(82.4)			
***Morel et al.***	2009	France	226(NR/NR)	56 (20–94)	<180	AITD	435(574.8)	588.75(972.59)	NR	NR	CLIA	NR NR	
						Hypothyroidism	435(574.8)	557(1001.4)	NR	NR			
***Mehmet et al.***	2012	Turkey	200(173/27)	44.9(14.2)	<189	Hypothyroidism	299.1 (205.5)	400.2 (314.5)	18(18)	82(82)	NR	NR	NR
						SH	299.1 (205.5)	348.5(211.8)	25(25)	75(75)			
***Caplan et al.***	1975	United States of America	103(NR/NR)	56.8 (2.1)	<200	Hypothyroidism	412 (213.12)	450 (231.98)	NR	NR	Microbiological assay	NR	NR
						Hyperthyroidism	412 (213.12)	499 (253.65)	NR	NR			
***Ranjan et al.***	2020	India	150(NR/NR)	NR(NR)	NR	Hypothyroidism	314.85 (41.1)	277.2 (37.89)	NR	NR	CLIA	NR	NR
						SH	314.85 (41.1)	277.2 (37.89)	NR	NR			
***Onat et al.***	2003	Turkey	85(74/11)	59.93 (19.84)	NR	Hypothyroidism	521.92 (121.45)	225.6 (97.16)	NR	NR	CLIA	NR	NR
						Hyperthyroidism	521.92 (121.45)	359.36 (323.15)	NR	NR			
***Photam et al.***	2014	India	47(NR/NR)	NR(NR)	NR	Hyperthyroidism	205(111.85)	249(165.18)	NR	NR	NR	NR	NR
						AITD	205(111.85)	249(165.18)	NR	NR			
***Miskiewicz et al.***	2015	Poland	8(5/3)	33 (22–68)	NR	AITD	NR	NR	2(25)	6(75)	NR	1(12.5)	7(87.5)
						Hyperthyroidism	NR	NR	2(25)	6(75)			
***Orzechowska-Pawilojc et al.***	2007	Poland	61(61/0)	37.9(10.3)	<179	AITD	420.83(142.07)	329.69(154.37)	3(9.7)	28(90.3)	CLIA	NR	NR
						Hypothyroidism	420.83(142.07)	329.69(154.37)	3(9.7)	28(90.3)			
***Nicolaou et al.***	2014	Greece	115(99/16)	47.7(12.9)	NR	AITD	NR	NR	19(16.5)	96(83.5)	NR	33(28.7)	82(71.3)
						Hypothyroidism	NR	NR	19(16.5)	96(83.5)			
***Aon et al.***	2022	Kuwait	93(80/13)	34(14)	<133	Hypothyroidism	NR	NR	15(33.3)	30(66.7)	CLIA	NR	NR
						SH	NR	NR	23(47.9)	25(52.1)			
***Aktaş et al.***	2020	Turkey	130(115/15)	41.4(1.9)	<200	AITD	NR	NR	60(46.15)	70(53.85)	CLIA	NR	NR
						Hypothyroidism	NR	NR	60(46.15)	70(53.85)			
***Nalbant et al.***	2016	Turkey	211(194/17)	39.31(11.44)	NR	AITD	261.5(109.3)	259(105.1)	51(24.17)	160(75.83)	CLIA	NR	NR
						Hyperthyroidism	261.5(109.3)	239(68.7)	NR	NR			
						Hypothyroidism	261.5(109.3)	249.3(85.9)	51(24.17)	160(75.83)			
***Erdal et al.***	2008	Turkey	43(39/4)	48.5(4.7)	<193	AITD	NR	NR	0(0)	43(100)	CLIA	NR	NR
						Hypothyroidism	NR	NR	0(0)	43(100)			
						SH	NR	NR	0(0)	43(100)			

AITD, autoimmune thyroid disease; APCA, anti-parietal cell antibodies; CLIA, chemiluminescence immunoassay; ECLIA, electrochemiluminescence immunoassay; FEI, fluorescence enzyme immunoassay; HPLC, high-performance liquid chromatography; IQR, interquartile range; NR, not reported; RIA, radio immunoassay; SH, subclinical hypothyroidism; SD, standard deviation; TD, thyroid disorder; 95% CI, 95% confidence interval.

We sent emails requesting the missing information to the authors, but received no reply. A total of 37 studies were classified as having a low risk of bias, and 27 as having a high risk of bias (**Supplemental Table S1**).

Of the studies included, 40 (n = 24835) evaluated vitamin B12 abnormalities in patients with hypothyroidism. Of these, 20 determined the frequency of vitamin B12 deficiency in patients with hypothyroidism, 16 determined the MD between the vitamin B12 levels of healthy participants and those with hypothyroidism, and 4 evaluated both the MD and the frequencies.

A total of 17 studies (n = 3795) evaluated the B12 levels in patients with hyperthyroidism. Of these, 7 evaluated the frequency of B12 deficiency in patients with hyperthyroidism. As well, 7 evaluated the MD in the B12 levels between healthy participants and patients with hyperthyroidism. The remaining 3 studies evaluated both the MD and the frequencies.

We found 21 studies (n = 4901) that evaluated B12 deficiencies in patients with AITD. Of these, 14 evaluated the frequency of B12 deficiency in AITD, 3 evaluated the MDs in the vitamin B12 levels between healthy participants and AITD patients, and 4 evaluated both the MDs and frequencies.

A total of 10 studies (n = 18712) evaluated B12 deficiencies in patients with SH. Of these, 3 assessed the frequency of B12 deficiency in SH, 6 evaluated the MDs in vitamin B12 levels between healthy participants and patients with SH, and only 1 evaluated both the MD and frequency.

### 3.3 Differences in the serum levels of vitamin B12 among patients with and without TD

#### 3.3.1 Differences between the vitamin B12 levels of patients with hypothyroidism and healthy participants

**Figure 2:**
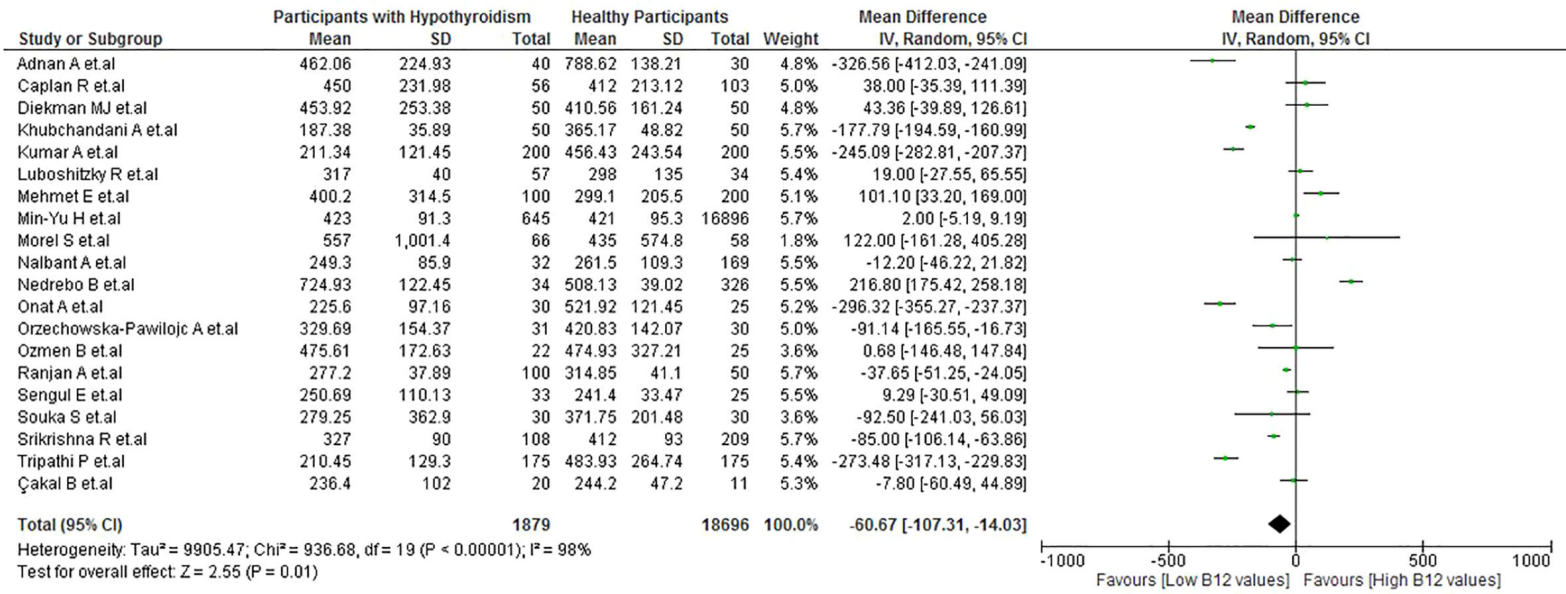
Vitamin B12 values in patients with hypothyroidism vs healthy patients.

Patients with hypothyroidism had lower B12 levels than healthy participants (MD: −60.67 pg/mL; 95% CI: −107.31 to −14.03 pg/mL; p = 0.01, I^2^ = 98%) (**Figure 2**). A subgroup analysis by continent (**Supplemental Figure S1**) revealed that, in Asian countries, the statistical significance of this difference remained with high heterogeneity (MD: −133.04 pg/mL; 95% CI: −197.84 to −68.23 pg/mL; P < 0.001, I^2^ = 99%); meanwhile, no statistical significance was observed in European countries (MD: 3.11 pg/mL; 95% CI: −88.39 to 94.62 pg/mL; p = 0.95). In the sensitivity analysis, after removing the studies with a high risk of bias, there was a decrease in heterogeneity (I^2 =^ 53%) (**Supplemental Figure S2**).

#### 3.3.2 Differences between vitamin B12 levels of patients with hyperthyroidism and healthy participants

**Figure 3:**
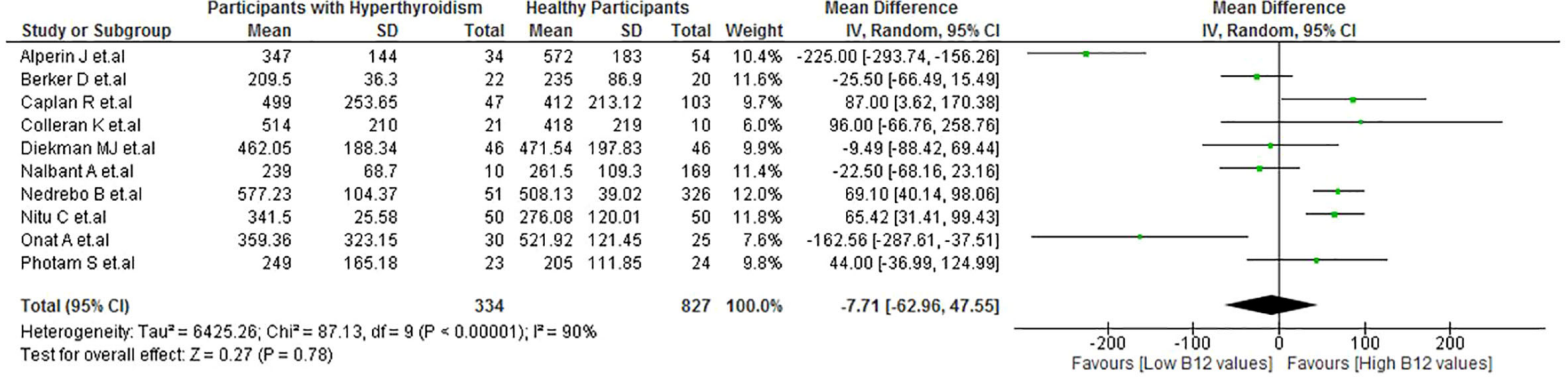
Vitamin B12 values in patients with hyperthyroidism vs healthy patients.

No significant difference was observed in the B12 levels between patients with hyperthyroidism and healthy participants (MD: −7.71 pg/mL; 95% CI: −62.96 to 47.55 pg/mL; p = 0.78, I^2 =^ 90%) (**Figure 3**). There were also no significant differences in the subgroups analysis (**Supplemental Figure S3**). In the sensitivity analysis, after removing the studies with a high risk of bias, there was a decrease in heterogeneity (I^2 =^ 0%) (**Supplemental Figure S4**).

#### 3.3.3 Differences between the vitamin B12 levels of patients with autoimmune thyroid disease and healthy participants

**Figure 4:**
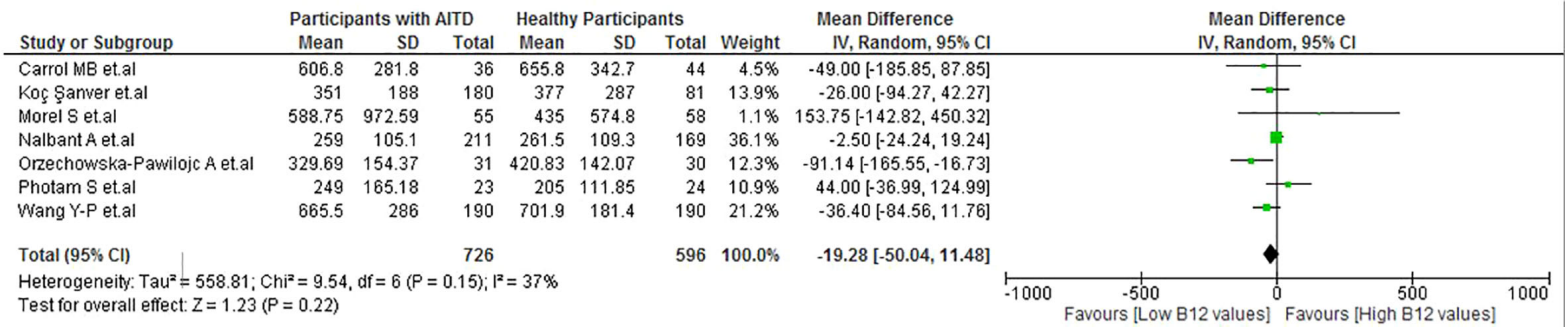
Vitamin B12 values in patients with AITD vs healthy participants.

No significant difference was observed in the B12 levels between patients with AITD and healthy participants (MD: −19.28 pg/mL; 95% CI: −50.04 to 11.48 pg/mL; p = 0.22, I^2^ = 37%) (**Figure 4**). As well, there were no significant differences in the subgroup analysis (**Supplemental Figure S5**). In the sensitivity analysis, after removing studies with a high risk of bias, low heterogeneity remained (I^2 =^ 33%) (**Supplemental Figure S6**).

#### 3.3.4 Differences between the vitamin B12 levels of patients with subclinical hypothyroidism and healthy participants

**Figure 5:**
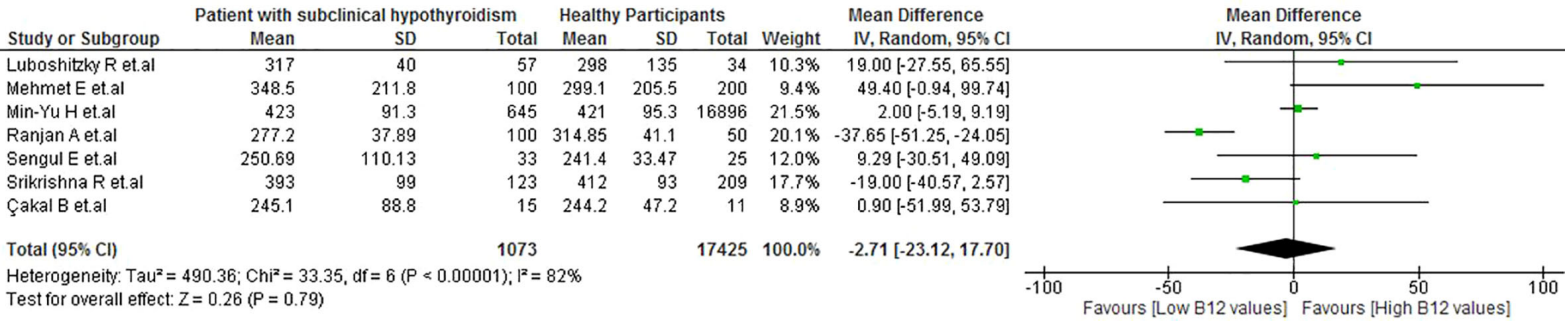
Vitamin B12 values in patients with SH vs healthy participants.

No significant differences in the levels of vitamin B12 were observed between healthy participants and patients with SH (MD: −2.71 pg/mL; 95% CI: −23.12 to 17.7 pg/mL; p = 0.79, I^2^ = 82%) (**Figure 5**). Also, there were no significant differences in the subgroup analysis (**Supplemental Figure S7**). Regarding the sensitivity analysis, after removing the studies with a high risk of bias, high heterogeneity remained (I^2 =^ 70%) (**Supplemental Figure S8**).

### 3.4 Frequency of vitamin B12 deficiency in patients with TD

#### 3.4.1 Evaluation of the frequency of vitamin B12 deficiency in hypothyroidism

The frequency of vitamin B12 deficiency in patients with hypothyroidism was 27.0% (95% CI: 19.0% to 36.0%), with high heterogeneity among studies (I^2^ = 97%) (**Supplemental Figure S9**). In the sensitivity analysis, after removing the studies with a high risk of bias, high heterogeneity remained (I^2^ = 96.5%) (**Supplemental Figure S10**).

#### 3.4.2 Evaluation of the frequency of vitamin B12 deficiency in hyperthyroidism

The frequency of B12 deficiency in patients with hyperthyroidism was 6.0% (95% CI: 2.0% to 11.0%) with moderate heterogeneity among studies (I^2 =^ 60%) (**Supplemental Figure S11**). In the sensitivity analysis, after removing the studies with a high risk of bias, there was a decrease in heterogeneity (I^2^ = 28.03%) (**Supplemental Figure S12**).

#### 3.4.3 Evaluation of the frequency of vitamin B12 deficiency in autoimmune thyroid disease

The frequency of vitamin B12 deficiency in patients with hypothyroidism was 18.0% (95% CI: 11.0% to 27.0%), with high heterogeneity among studies (I^2^ = 97%) (**Supplemental Figure S13**). In the sensitivity analysis, after removing the studies with a high risk of bias, high heterogeneity remained (I^2^ = 97%) (**Supplemental Figure S14**).

#### 3.4.4 Evaluation of the frequency of vitamin B12 deficiency in subclinical hypothyroidism

The frequency of vitamin B12 deficiency in patients with SH was 27.0% (95% CI: 5.0% to 57.0%), with high heterogeneity among studies (I^2^ = 95%) (**Supplemental Figure S15**).

### 3.5 Anti-parietal cell antibodies and autoimmune thyroid disease

**Figure 6:**
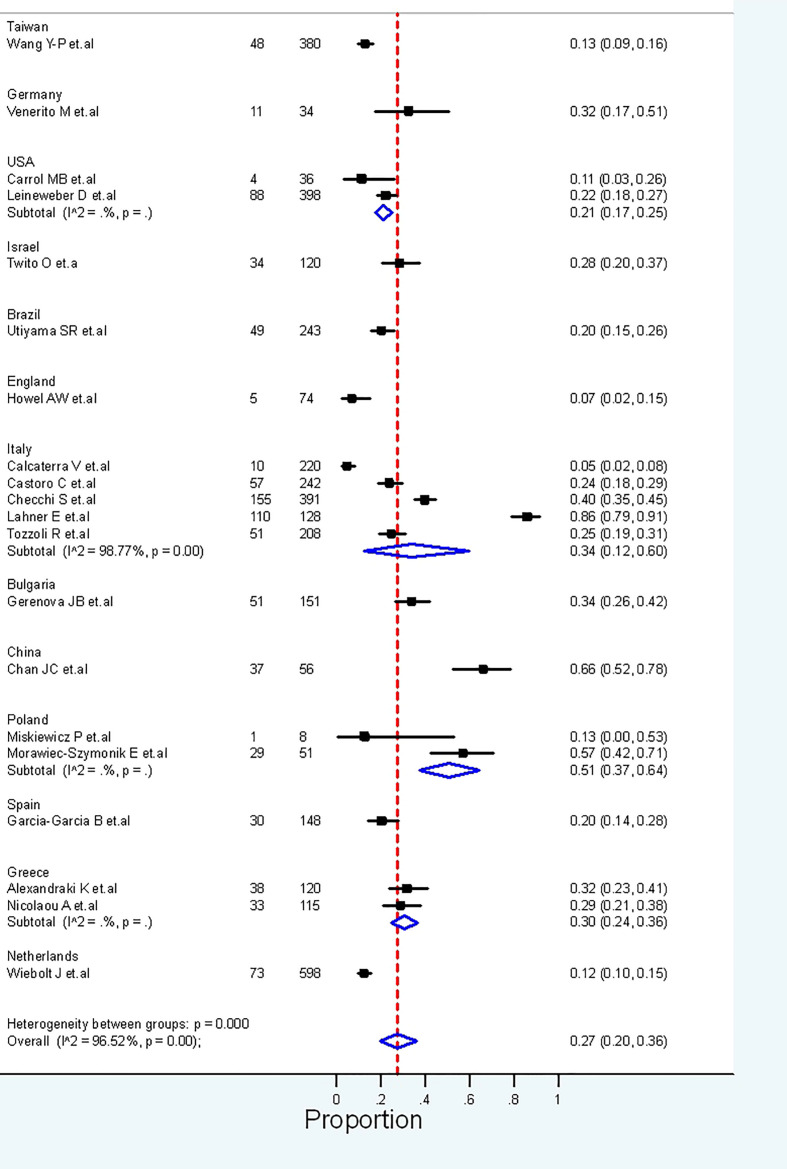
Frequency of the presence of APCA in AITD.

The frequency of APCA in patients with AITD was evaluated in 20 studies (n = 3721). Overall, these studies found APCA to be present in 27.0% (95% CI: 20.0% to 36.0%) of patients with AITD, with high heterogeneity between studies (I2 = 96%) (**Figure 6**). Regarding the sensitivity analysis, after removing the studies with a high risk of bias, high heterogeneity remained (I2 = 98.19%) (**Supplemental Figure S16**).

### 3.6 Publication bias

When using the Egger test, no publication bias was found in the evaluations of vitamin B12 levels in hypothyroidism (p = 0.495), hyperthyroidism (p = 0.632), AITD (p = 0.687), and SH (p = 0.159). A funnel plot showed no asymmetry in any of the scenarios (**Supplemental Figures S17**-**S20**).

## 4 Discussion

The main finding of our study was the significant difference in the vitamin B12 levels between patients with hypothyroidism and healthy participants. However, no significant difference was observed in the vitamin B12 levels between patients with hyperthyroidism/AITD/SH and healthy participants. The frequencies of vitamin B12 deficiency in patients with SH, hypothyroidism, hyperthyroidism, and AITD were 27%, 27%, 6%, and 18%, respectively, and the frequency of APCA in AITD was 27%. Our results should serve as a basis for the modification of clinical practice guidelines on TD.

Vitamin B12 is synthesized by intestinal anaerobic microorganisms, albeit its impact is still unexplored; while oral dietary intake provides 50% of B12 requirements (83, 84). Vitamin B12 is naturally found in foods of animal origin and in crystalline form in supplements and fortified foods. It comes in the form of cyanocobalamin or hydroxocobalamin, which can be converted to either of the two forms of vitamin B12 cofactors, whose transformation reactions are essential for the synthesis of nucleic acids, myelination of the nerves and axons of the central nervous system, and efficient bone marrow erythropoiesis (15). Thus, an adequate supply of this vitamin is required to maintain these biological processes.

Inadequate dietary intake and malabsorption are the major causes of vitamin B12 deficiency (83), though the first rarely occurs in high-income countries, as foods of animal origin are an important component of the diet. Nevertheless, it may occur in strict vegetarians and malnourished older adults (85). On the other hand, B12 malabsorption is found in some medical conditions such as AAG, *Helicobacter pylori* infection, PA, and long-term antacid treatment (15). It can also be a consequence of surgeries such as partial gastrectomy and gastric bypass (15).

Previously, a narrative review found a frequency of B12 deficiency ranging from 10% to 40.5% in patients with hypothyroidism and from 6.3% to 55.5% in patients with AITD (15). However, it was not as comprehensive as our systematic review, in which we conducted a meta-analysis. Our results were based on a larger number of studies, thus decreasing variations and generating a more accurate frequency. Nonetheless, the prevalence of B12 deficiency varies depending on the cut-off used to define it. The B12 level that constitutes deficiency depends on both the population and the method employed to measure the B12 levels. For instance, serum vitamin B12 levels < 148 pmol/L are generally considered deficient in high-income countries. Using this parameter, vitamin B12 deficiency has been shown to increase with age, from 3% in young adults to 10% in older adults (82). The incidence of subclinical B12 deficiency, defined as serum vitamin B12 levels of 148–221 pmol/L, affects about 20% of older adults (82). Contrarily, the incidence is higher in low-income countries, where low and borderline B12 levels are detected in around 70% of adults (82). It is important to note that serum B12 is the primary test used in clinical practice, in spite of its poor sensitivity and specificity for identifying B12 deficiency (82). Although more sensitive tests have been developed, including plasma methylmalonic acid (MMA), homocysteine, and serum holotranscobalamin, they are expensive and not routinely available. Moreover, they do not have defined cut-off points to denote deficiency. Accordingly, their role in clinical practice is still unclear (86).

The association between vitamin B12 deficiency and TD has been studied with a particular focus on patients with AITD (15). AITD encompasses a group of disorders characterized by the production of antibodies against the thyroid gland, with Graves’ disease and Hashimoto’s thyroiditis being the most common (15). Even though the causes of vitamin B12 deficiency among these patients are likely to be multifactorial, they would be predominantly related to the comorbidity of other autoimmune disorders, such as AAG, PA (10), and celiac disease (11). Indeed, the frequency of AAG among patients with AITD ranges from 35% to 40%; meanwhile, the frequency of PA in the same group of patients reaches 16% (53, 87). As well, 26% of patients with celiac disease also have AITD (11).

In the absence of AITD, the causes of vitamin B12 deficiency in those with hypothyroidism have been studied in less detail; however, they might be related to dietary habits (12). Alterations in the composition of the microbiota, bacterial overgrowth, and slow intestinal motility have also been proposed as potential causes in these patients (8, 12). Given the increased frequency of hypothyroidism and vitamin B12 deficiency with ageing, age should also be considered as a contributing factor (5, 88). This convergence of factors would explain not only the vitamin B12 deficiency but also the deficiencies in trace elements, which are also frequently evidenced in patients with hypothyroidism and SH (89). In a meta-analysis carried out in India, the frequency of B12 deficiency in healthy adults was 48% (90), being higher than the frequencies of all the TDs we obtained.

While tests for the presence of APCA are considered the most sensitive form of AAG diagnosis (91), some studies have suggested that these antibodies can also be found in 7.8% of healthy individuals and 19.5% of patients infected with H. pylori (14, 91). Their association with other autoimmune diseases, such as type 1 diabetes mellitus, vitiligo, and celiac disease, is also well known (26, 92). Several studies have demonstrated that APCA-positive patients have a higher incidence of anemia as the involved antibodies can induce the destruction of gastric parietal cells, preventing the production of intrinsic factor, and leading to insufficient vitamin B12 absorption and PA (47, 48, 93). Variations in the frequency of APCA would differ due to the heterogeneity of the studies, but some researchers have suggested that this variation may also be attributable to the AITD type. Utiyama et al. found that 20.16% of patients with AITD tested positive for APCA, with a frequency of 21.3% among those with Graves’ disease and 18.6% among those with Hashimoto’s thyroiditis (48).

Our findings are particularly useful in clinical settings as they emphasize the necessity of in-depth evaluations of vitamin B12 levels in patients with TD. Nearly one in four patients with either SH or hypothyroidism suffers from B12 deficiency. Although it is tempting to suggest routine vitamin B12 assessment in patients with TD, more studies are needed to support this practice. There is still scarce evidence suggesting that the administration of vitamins with antioxidant properties in patients with TD, such as hyperthyroidism, can decrease the severity of clinical symptoms (94). Likewise, some studies suggest vitamin D supplementation can have a beneficial effect on bone system among these patients (94). Nevertheless, the role of vitamin D is controversial. A systematic review revealed that although there are various health benefits of dietary supplements in the prevention and treatment of several TD, there are also many risks associated with the use of these supplements (95). In this regard, clinical practice guidelines should include nutritional assessments as part of the management of TD patients. We found that many of the current guidelines on TD do not require a comprehensive nutritional evaluation as part of their management plan, nor do they recommend assessing and addressing B12 deficiencies (96–98).

### 4.1 Limitations

This study has some limitations that need to be considered. Firstly, most of the studies included were conducted on the Asian continent, with few were from other continents. Differences in B12 levels among people of different ethnicities should be measured and compared to determine whether the results of this review can be ethnically generalised. Secondly, there was high statistical heterogeneity caused by clinical and methodological differences, and our sensitivity analysis was only able to reduce it in some cases. Thirdly, there were few studies assessing the association between B12 vitamin and the levels of TPO-Ab and Tg-Ab. We therefore encourage further evaluation of this association in future studies. Finally, vitamin B12 levels were not adjusted for sociodemographic variables or comorbidities. Such adjustment could allow a cut-off consensus to be obtained according to the conditions of each population and should be considered in future studies.

## 5 Conclusion

Patients with hypothyroidism had lower levels of vitamin B12 than healthy participants. No significant differences were found between vitamin B12 levels and hyperthyroidism, AITD, or SH. The co-occurrence of APCA and vitamin B12 deficiency in TD patients did not exceed 30% in any of the reviewed studies.

## Data availability statement

The original contributions presented in the study are included in the article/**Supplementary Material**. Further inquiries can be directed to the corresponding author.

## Author contributions

Conceptualization, VB-Z, JU-B, EA-B, EH-B, AA-C, PH-A and FI-C. Data curation, VB-Z, JU-B, EA-B and EH-B. Formal analysis, JU-B, EA-B and VB-Z. Methodology, VB-Z, JU-B, EA-B, and EH-B. Writing original draft, VB-Z, JU-B, EA-B, EH-B, AA-C, PH-A and FI-C. Review and editing, JU-B, AA-C, FI-C, PH-A and VB-Z. All authors contributed to the article and approved the submitted version.
