# Supplementary material for: Vitamin B12 levels in thyroid disorders: A systematic review and meta-analysis
Source: Front Endocrinol (Lausanne). 2023 Feb 22;14:1070592. doi: 10.3389/fendo.2023.1070592 (PMC9994182; doi:10.3389/fendo.2023.1070592)
Supplement: Supplementary file 1 [file DataSheet_1.docx]

Supplementary Material

**Vitamin B12 levels in thyroid disorders: A systematic review and meta-analysis**

**Appendix 1. Search Strategy**

**PubMed**

**#1:** “Vitamin B 12 Deficiency” [MH] OR ((“vit*” [TIAB] AND “B12” [TIAB]) AND “deficienc*” [TIAB]) OR ((“vit*” [TIAB] AND (“B” [TIAB] AND “12” [TIAB])) AND “deficienc*” [TIAB]) OR (“deficienc*” [TIAB] AND “B12” [TIAB]) OR (“deficienc*” [TIAB] AND (“B” [TIAB] AND “12” [TIAB])) OR (“B12” [TIAB] AND “deficienc*” [TIAB]) OR ((“B” [TIAB] AND “12” [TIAB]) AND “deficienc*” [TIAB]) OR “acobalaminosis” [TIAB] OR “AdCbl deficienc*” [TIAB] OR “adenosylcobalamin deficienc*” [TIAB] OR “AdoCbl deficienc*” [TIAB] OR (“avitaminosis” [TIAB] AND “B12” [TIAB]) OR (“avitaminosis” [TIAB] AND (“B” [TIAB] AND “12” [TIAB])) OR (“hypovitaminosis” [TIAB] AND “B12” [TIAB]) OR (“hypovitaminosis” [TIAB] AND (“B” [TIAB] AND “12” [TIAB])) OR (“hypovitaminosis” [TIAB] AND “B12” [TIAB]) OR (“hypovitaminosis” [TIAB] AND (“B” [TIAB] AND “12” [TIAB])) OR ((“B12” [TIAB] AND “vit*” [TIAB]) AND “deficienc*” [TIAB]) OR (((“B” [TIAB] AND “12” [TIAB]) AND “vit*” [TIAB]) AND “deficienc*” [TIAB]) OR “Cbl deficienc*” [TIAB] OR “CN-Cbl deficienc*” [TIAB] OR “cobalamin deficienc*” [TIAB] OR “cyanocobalamin deficienc*” [TIAB] OR “hypocobalaminosis” [TIAB] OR “hypocyanocobalaminosis” [TIAB] OR “MeCbl deficienc*” [TIAB] OR “methylcobalamin deficienc*” [TIAB] OR ((“vit*” [OT] AND “B12” [OT]) AND “deficienc*” [OT]) OR ((“vit*” [OT] AND (“B” [OT] AND “12” [OT])) AND “deficienc*” [OT]) OR (“deficienc*” [OT] AND “B12” [OT]) OR (“deficienc*” [OT] AND (“B” [OT] AND “12” [OT])) OR (“B12” [OT] AND “deficienc*” [OT]) OR ((“B” [OT] AND “12” [OT]) AND “deficienc*” [OT]) OR “acobalaminosis” [OT] OR “AdCbl deficienc*” [OT] OR “adenosylcobalamin deficienc*” [OT] OR “AdoCbl deficienc*” [OT] OR (“avitaminosis” [OT] AND “B12” [OT]) OR (“avitaminosis” [OT] AND (“B” [OT] AND “12” [OT])) OR (“hypovitaminosis” [OT] AND “B12” [OT]) OR (“hypovitaminosis” [OT] AND (“B” [OT] AND “12” [OT])) OR (“hypovitaminosis” [OT] AND “B12” [OT]) OR (“hypovitaminosis” [OT] AND (“B” [OT] AND “12” [OT])) OR ((“B12” [OT] AND “vit*” [OT]) AND “deficienc*” [OT]) OR (((“B” [OT] AND “12” [OT]) AND “vit*” [OT]) AND “deficienc*” [OT]) OR “Cbl deficienc*” [OT] OR “CN-Cbl deficienc*” [OT] OR “cobalamin deficienc*” [OT] OR “cyanocobalamin deficienc*” [OT] OR “hypocobalaminosis” [OT] OR “hypocyanocobalaminosis” [OT] OR “MeCbl deficienc*” [OT] OR “methylcobalamin deficienc*” [OT]

**#2:** Anemia, Pernicious [MH] OR (“addison*” [TIAB] AND “anaemia” [TIAB]) OR (“addison*” [TIAB] AND “anemia” [TIAB]) OR (“anemia” [TIAB] AND “pernicio*” [TIAB]) OR (“anaemia” [TIAB] AND “pernicio*” [TIAB]) OR (“pernicio*” [TIAB] AND “anemia” [TIAB]) OR (“pernicio*” [TIAB] AND “anaemia” [TIAB]) OR (“biermer” [TIAB] AND “anaemia” [TIAB]) OR (“biermer” [TIAB] AND “anemia” [TIAB]) OR (“biermer” [TIAB] AND “disease” [TIAB]) OR (“addison*” [OT] AND “anaemia” [OT]) OR (“addison*” [OT] AND “anemia” [OT]) OR (“anemia” [OT] AND “pernicio*” [OT]) OR (“anaemia” [OT] AND “pernicio*” [OT]) OR (“pernicio*” [OT] AND “anemia” [OT]) OR (“pernicio*” [OT] AND “anaemia” [OT]) OR (“biermer” [OT] AND “anaemia” [OT]) OR (“biermer” [OT] AND “anemia” [OT]) OR (“biermer” [OT] AND “disease” [OT])

**#3:** Thyroid Diseases [MH] OR (“thyroid*” [TIAB] AND “abnormalit*” [TIAB]) OR (“thyroid*” [TIAB] AND “anomal*” [TIAB]) OR (“thyroid*” [TIAB] AND “disease*” [TIAB]) OR (“thyroid*” [TIAB] AND “disorder*” [TIAB]) OR (“thyroid*” [TIAB] AND “dysfunction*” [TIAB]) OR (“thyroid*” [OT] AND “abnormalit*” [OT]) OR (“thyroid*” [OT] AND “anomal*” [OT]) OR (“thyroid*” [OT] AND “disease*” [OT]) OR (“thyroid*” [OT] AND “disorder*” [OT]) OR (“thyroid*” [OT] AND “dysfunction*” [OT])

**#4:** #1 OR #2

**#5:** #3 AND #4

**Scopus**

**#1:** TIAB=(“Vitamin B 12 Deficiency” OR ((“vit*” W/0 “B12”) W/3 “deficienc*”) OR ((“vit*” W/0 (“B” W/0 “12”)) W/3 “deficienc*”) OR (“deficienc*” W/0 “B12”) OR (“deficienc*” W/0 (“B” W/0 “12”)) OR (“B12” W/0 “deficienc*”) OR ((“B” W/0 “12”) W/0 “deficienc*”) OR “acobalaminosis” OR “AdCbl deficienc*” OR “adenosylcobalamin deficienc*” OR “AdoCbl deficienc*” OR (“avitaminosis” W/0 “B12”) OR (“avitaminosis” W/0 (“B” W/0 “12”)) OR (“hypovitaminosis” W/0 “B12”) OR (“hypovitaminosis” W/0 (“B” W/0 “12”)) OR (“hypovitaminosis” W/0 “B12”) OR (“hypovitaminosis” W/0 (“B” W/0 “12”)) OR ((“B12” W/0 “vit*”) W/3 “deficienc*”) OR (((“B” W/0 “12”) W/0 “vit*”) W/3 “deficienc*”) OR “Cbl deficienc*” OR “CN-Cbl deficienc*” OR “cobalamin deficienc*” OR “cyanocobalamin deficienc*” OR “hypocobalaminosis” OR “hypocyanocobalaminosis” OR “MeCbl deficienc*” OR “methylcobalamin deficienc*”)

**#2**: TIAB=(“Anemia, Pernicious” OR (“addison*” W/3 “anaemia”) OR (“addison*” W/3 “anemia”) OR (“anemia” W/0 “pernicio*”) OR (“anaemia” W/0 “pernicio*”) OR (“pernicio*” W/0 “anemia”) OR (“pernicio*” W/0 “anaemia”) OR (“biermer” W/0 “anaemia”) OR (“biermer” W/0 “anemia”) OR (“biermer” W/0 “disease”))

**#3:** TIAB=(“Thyroid Diseases” OR (“thyroid*” W/0 “abnormalit*”) OR (“thyroid*” W/0 “anomal*”) OR (“thyroid*” W/1 “disease*”) OR (“thyroid*” W/0 “disorder*”) OR (“thyroid*” W/1 “dysfunction*”))

**#4:** #1 OR #2

**#5:** #3 AND #4

**WoS**

**#1:** AK=(“Vitamin B 12 Deficiency” OR ((“vit*” NEAR/0 “B12”) NEAR/3 “deficienc*”) OR ((“vit*” NEAR/0 (“B” NEAR/0 “12”)) NEAR/3 “deficienc*”) OR (“deficienc*” NEAR/0 “B12”) OR (“deficienc*” NEAR/0 (“B” NEAR/0 “12”)) OR (“B12” NEAR/0 “deficienc*”) OR ((“B” NEAR/0 “12”) NEAR/0 “deficienc*”) OR “acobalaminosis” OR “AdCbl deficienc*” OR “adenosylcobalamin deficienc*” OR “AdoCbl deficienc*” OR (“avitaminosis” NEAR/0 “B12”) OR (“avitaminosis” NEAR/0 (“B” NEAR/0 “12”)) OR (“hypovitaminosis” NEAR/0 “B12”) OR (“hypovitaminosis” NEAR/0 (“B” NEAR/0 “12”)) OR (“hypovitaminosis” NEAR/0 “B12”) OR (“hypovitaminosis” NEAR/0 (“B” NEAR/0 “12”)) OR ((“B12” NEAR/0 “vit*”) NEAR/3 “deficienc*”) OR (((“B” NEAR/0 “12”) NEAR/0 “vit*”) NEAR/3 “deficienc*”) OR “Cbl deficienc*” OR “CN-Cbl deficienc*” OR “cobalamin deficienc*” OR “cyanocobalamin deficienc*” OR “hypocobalaminosis” OR “hypocyanocobalaminosis” OR “MeCbl deficienc*” OR “methylcobalamin deficienc*”) OR KP=(“Vitamin B 12 Deficiency” OR ((“vit*” NEAR/0 “B12”) NEAR/3 “deficienc*”) OR ((“vit*” NEAR/0 (“B” NEAR/0 “12”)) NEAR/3 “deficienc*”) OR (“deficienc*” NEAR/0 “B12”) OR (“deficienc*” NEAR/0 (“B” NEAR/0 “12”)) OR (“B12” NEAR/0 “deficienc*”) OR ((“B” NEAR/0 “12”) NEAR/0 “deficienc*”) OR “acobalaminosis” OR “AdCbl deficienc*” OR “adenosylcobalamin deficienc*” OR “AdoCbl deficienc*” OR (“avitaminosis” NEAR/0 “B12”) OR (“avitaminosis” NEAR/0 (“B” NEAR/0 “12”)) OR (“hypovitaminosis” NEAR/0 “B12”) OR (“hypovitaminosis” NEAR/0 (“B” NEAR/0 “12”)) OR (“hypovitaminosis” NEAR/0 “B12”) OR (“hypovitaminosis” NEAR/0 (“B” NEAR/0 “12”)) OR ((“B12” NEAR/0 “vit*”) NEAR/3 “deficienc*”) OR (((“B” NEAR/0 “12”) NEAR/0 “vit*”) NEAR/3 “deficienc*”) OR “Cbl deficienc*” OR “CN-Cbl deficienc*” OR “cobalamin deficienc*” OR “cyanocobalamin deficienc*” OR “hypocobalaminosis” OR “hypocyanocobalaminosis” OR “MeCbl deficienc*” OR “methylcobalamin deficienc*”) OR TI=(“Vitamin B 12 Deficiency” OR ((“vit*” NEAR/0 “B12”) NEAR/3 “deficienc*”) OR ((“vit*” NEAR/0 (“B” NEAR/0 “12”)) NEAR/3 “deficienc*”) OR (“deficienc*” NEAR/0 “B12”) OR (“deficienc*” NEAR/0 (“B” NEAR/0 “12”)) OR (“B12” NEAR/0 “deficienc*”) OR ((“B” NEAR/0 “12”) NEAR/0 “deficienc*”) OR “acobalaminosis” OR “AdCbl deficienc*” OR “adenosylcobalamin deficienc*” OR “AdoCbl deficienc*” OR (“avitaminosis” NEAR/0 “B12”) OR (“avitaminosis” NEAR/0 (“B” NEAR/0 “12”)) OR (“hypovitaminosis” NEAR/0 “B12”) OR (“hypovitaminosis” NEAR/0 (“B” NEAR/0 “12”)) OR (“hypovitaminosis” NEAR/0 “B12”) OR (“hypovitaminosis” NEAR/0 (“B” NEAR/0 “12”)) OR ((“B12” NEAR/0 “vit*”) NEAR/3 “deficienc*”) OR (((“B” NEAR/0 “12”) NEAR/0 “vit*”) NEAR/3 “deficienc*”) OR “Cbl deficienc*” OR “CN-Cbl deficienc*” OR “cobalamin deficienc*” OR “cyanocobalamin deficienc*” OR “hypocobalaminosis” OR “hypocyanocobalaminosis” OR “MeCbl deficienc*” OR “methylcobalamin deficienc*”) OR TS=(“Vitamin B 12 Deficiency” OR ((“vit*” NEAR/0 “B12”) NEAR/3 “deficienc*”) OR ((“vit*” NEAR/0 (“B” NEAR/0 “12”)) NEAR/3 “deficienc*”) OR (“deficienc*” NEAR/0 “B12”) OR (“deficienc*” NEAR/0 (“B” NEAR/0 “12”)) OR (“B12” NEAR/0 “deficienc*”) OR ((“B” NEAR/0 “12”) NEAR/0 “deficienc*”) OR “acobalaminosis” OR “AdCbl deficienc*” OR “adenosylcobalamin deficienc*” OR “AdoCbl deficienc*” OR (“avitaminosis” NEAR/0 “B12”) OR (“avitaminosis” NEAR/0 (“B” NEAR/0 “12”)) OR (“hypovitaminosis” NEAR/0 “B12”) OR (“hypovitaminosis” NEAR/0 (“B” NEAR/0 “12”)) OR (“hypovitaminosis” NEAR/0 “B12”) OR (“hypovitaminosis” NEAR/0 (“B” NEAR/0 “12”)) OR ((“B12” NEAR/0 “vit*”) NEAR/3 “deficienc*”) OR (((“B” NEAR/0 “12”) NEAR/0 “vit*”) NEAR/3 “deficienc*”) OR “Cbl deficienc*” OR “CN-Cbl deficienc*” OR “cobalamin deficienc*” OR “cyanocobalamin deficienc*” OR “hypocobalaminosis” OR “hypocyanocobalaminosis” OR “MeCbl deficienc*” OR “methylcobalamin deficienc*”)

**#2:**

AK=(“Anemia, Pernicious” OR (“addison*” NEAR/3 “anaemia”) OR (“addison*” NEAR/3 “anemia”) OR (“anemia” NEAR/0 “pernicio*”) OR (“anaemia” NEAR/0 “pernicio*”) OR (“pernicio*” NEAR/0 “anemia”) OR (“pernicio*” NEAR/0 “anaemia”) OR (“biermer” NEAR/0 “anaemia”) OR (“biermer” NEAR/0 “anemia”) OR (“biermer” NEAR/0 “disease”)) OR KP=(“Anemia, Pernicious” OR (“addison*” NEAR/3 “anaemia”) OR (“addison*” NEAR/3 “anemia”) OR (“anemia” NEAR/0 “pernicio*”) OR (“anaemia” NEAR/0 “pernicio*”) OR (“pernicio*” NEAR/0 “anemia”) OR (“pernicio*” NEAR/0 “anaemia”) OR (“biermer” NEAR/0 “anaemia”) OR (“biermer” NEAR/0 “anemia”) OR (“biermer” NEAR/0 “disease”)) OR TI=(“Anemia, Pernicious” OR (“addison*” NEAR/3 “anaemia”) OR (“addison*” NEAR/3 “anemia”) OR (“anemia” NEAR/0 “pernicio*”) OR (“anaemia” NEAR/0 “pernicio*”) OR (“pernicio*” NEAR/0 “anemia”) OR (“pernicio*” NEAR/0 “anaemia”) OR (“biermer” NEAR/0 “anaemia”) OR (“biermer” NEAR/0 “anemia”) OR (“biermer” NEAR/0 “disease”)) OR TS=(“Anemia, Pernicious” OR (“addison*” NEAR/3 “anaemia”) OR (“addison*” NEAR/3 “anemia”) OR (“anemia” NEAR/0 “pernicio*”) OR (“anaemia” NEAR/0 “pernicio*”) OR (“pernicio*” NEAR/0 “anemia”) OR (“pernicio*” NEAR/0 “anaemia”) OR (“biermer” NEAR/0 “anaemia”) OR (“biermer” NEAR/0 “anemia”) OR (“biermer” NEAR/0 “disease”))

**#3:** AK=(“Thyroid Diseases” OR (“thyroid*” NEAR/0 “abnormalit*”) OR (“thyroid*” NEAR/0 “anomal*”) OR (“thyroid*” NEAR/1 “disease*”) OR (“thyroid*” NEAR/0 “disorder*”) OR (“thyroid*” NEAR/1 “dysfunction*”)) OR KP=(“Thyroid Diseases” OR (“thyroid*” NEAR/0 “abnormalit*”) OR (“thyroid*” NEAR/0 “anomal*”) OR (“thyroid*” NEAR/1 “disease*”) OR (“thyroid*” NEAR/0 “disorder*”) OR (“thyroid*” NEAR/1 “dysfunction*”)) OR TI=(“Thyroid Diseases” OR (“thyroid*” NEAR/0 “abnormalit*”) OR (“thyroid*” NEAR/0 “anomal*”) OR (“thyroid*” NEAR/1 “disease*”) OR (“thyroid*” NEAR/0 “disorder*”) OR (“thyroid*” NEAR/1 “dysfunction*”)) OR TS=(“Thyroid Diseases” OR (“thyroid*” NEAR/0 “abnormalit*”) OR (“thyroid*” NEAR/0 “anomal*”) OR (“thyroid*” NEAR/1 “disease*”) OR (“thyroid*” NEAR/0 “disorder*”) OR (“thyroid*” NEAR/1 “dysfunction*”))

**#4:** #1 OR #2

**#5:** #3 AND #4

**Embase**

**#1:** Vitamin B 12 Deficiency OR ((vit* NEAR/0 B12) NEAR/3 deficienc*) OR ((vit* NEAR/0 (B NEAR/0 12)) NEAR/3 deficienc*) OR (deficienc* NEAR/0 B12) OR (deficienc* NEAR/0 (B NEAR/0 12)) OR (B12 NEAR/0 deficienc*) OR ((B NEAR/0 12) NEAR/0 deficienc*) OR acobalaminosis OR AdCbl deficienc* OR adenosylcobalamin deficienc* OR AdoCbl deficienc* OR (avitaminosis NEAR/0 B12) OR (avitaminosis NEAR/0 (B NEAR/0 12)) OR (hypovitaminosis NEAR/0 B12) OR (hypovitaminosis NEAR/0 (B NEAR/0 12)) OR (hypovitaminosis NEAR/0 B12) OR (hypovitaminosis NEAR/0 (B NEAR/0 12)) OR ((B12 NEAR/0 vit*) NEAR/3 deficienc*) OR (((B NEAR/0 12) NEAR/0 vit*) NEAR/3 deficienc*) OR Cbl deficienc* OR CN-Cbl deficienc* OR cobalamin deficienc* OR cyanocobalamin deficienc* OR hypocobalaminosis OR hypocyanocobalaminosis OR MeCbl deficienc* OR methylcobalamin deficienc*

**#2:** Anemia, Pernicious OR (addison* NEAR/3 anaemia) OR (addison* NEAR/3 anemia) OR (anemia NEAR/0 pernicio*) OR (anaemia NEAR/0 pernicio*) OR (pernicio* NEAR/0 anemia) OR (pernicio* NEAR/0 anaemia) OR (biermer NEAR/0 anaemia) OR (biermer NEAR/0 anemia) OR (biermer NEAR/0 disease)

**#3:** Thyroid Diseases OR (thyroid* NEAR/0 abnormalit*) OR (thyroid* NEAR/0 anomal*) OR (thyroid* NEAR/1 disease*) OR (thyroid* NEAR/0 disorder*) OR (thyroid* NEAR/1 dysfunction*)

**#4:** #1 OR #2

**#5:** #3 AND #4

**Table S1. Quality assessment of included studies**

|  | **NEWCASTLE - OTTAWA QUALITY ASSESSMENT SCALE FOR CROSS-SECTIONAL STUDIES** | | | | | | | | | | |
| --- | --- | --- | --- | --- | --- | --- | --- | --- | --- | --- | --- |
| **STUDY** | | **SELECTION** | | | | **COMPARABILITY** | **OUTCOME** | | |  |  |
|  | | **Representativeness of the sample** | **Sample size** | **Non-respondents** | **Ascertainment of the exposure (risk factor)** | **The subjects in different outcome groups are comparable, based on the study design or analysis. Confounding factors are controlled.**  **Maximum : ☆☆** | **Assessment of outcome** | **Statistical test** | | **SCORE** | **Evidence quality** |
| **Das C et.al** | | ☆ | ☆ | ☆ | ☆ | ☆ | ☆ |  | | *6* | High Risk of Bias |
| **Bhuta P et.al** | | ☆ | ☆ | ☆ | ☆ | ☆ | ☆ |  | | *6* | High Risk of Bias |
| **Velarde-Mayol C et.al** | | ☆ | ☆ | ☆ | ☆ | ☆☆ | ☆ | ☆ | | *8* | Low risk of bias |
| **Calcaterra V et.al** | | ☆ | ☆ | ☆ | ☆ | ☆ | ☆ | ☆ | | *7* | Low risk of bias |
| **Dagdelen I et.al** | | ☆ | ☆ | ☆ | ☆ |  | ☆ |  | | *5* | High Risk of Bias |
| **Leineweber D et.al** | | ☆ | ☆ | ☆ | ☆ | ☆ | ☆ |  | | *6* | High Risk of Bias |
| **Wiebolt J et.al** | | ☆ | ☆ | ☆ | ☆ | ☆ | ☆ | ☆ | | *7* | Low risk of bias |
| **Sattar-Lakho A et.al** | | ☆ | ☆ | ☆ | ☆ | ☆☆ | ☆ | ☆ | | *8* | High Risk of Bias |
| **Jaya Kumari et.al** | | ☆ | ☆ | ☆ | ☆ | ☆☆ | ☆ | ☆ | | *8* | Low risk of bias |
| **Miskiewicz P et.al** | | ☆ | ☆ | ☆ | ☆ | ☆ | ☆ | ☆ | | *7* | Low risk of bias |
| **Orzechowska-Pawilojc A et.al** | | ☆ | ☆ | ☆ | ☆ | ☆☆ | ☆ | ☆ | | *8* | Low risk of bias |
| **Raju P et.al** | | ☆ | ☆ | ☆ | ☆ |  | ☆ |  | | *5* | High Risk of Bias |
| **Nicolaou A et.al** | | ☆ | ☆ | ☆ | ☆ | ☆ | ☆ | ☆ | | *7* | Low risk of bias |
| **Ness-Abramof R et.al** | | ☆ | ☆ | ☆ | ☆ | ☆ | ☆ | ☆ | | *7* | Low risk of bias |
| **Carrol MB et.al** | | ☆ | ☆ | ☆ | ☆ | ☆ | ☆ | ☆ | | *7* | Low risk of bias |
| **Siddique M et.al** | | ☆ | ☆ | ☆ | ☆ | ☆ | ☆ |  | | *6* | High Risk of Bias |
| **Aon M et.al** | | ☆ | ☆ | ☆ | ☆ | ☆☆ | ☆ | ☆ | | *8* | Low risk of bias |
| **Aktaş H et.al** | | ☆ | ☆ | ☆ | ☆ | ☆ | ☆ | ☆ | | *7* | Low risk of bias |
| **Jabbar A et.al** | | ☆ | ☆ | ☆ | ☆ |  | ☆ |  | | *5* | High Risk of Bias |
| **Jabeen A et.al** | | ☆ | ☆ | ☆ | ☆ | ☆ | ☆ | ☆ | | *7* | Low risk of bias |
| **Erdal M et.al** | | ☆ | ☆ | ☆ | ☆ | ☆☆ | ☆ | ☆ | | *8* | Low risk of bias |
| **Utiyama SR et.al** | | ☆ | ☆ | ☆ | ☆ | ☆☆ | ☆☆ | ☆ | | *9* | Low risk of bias |
| **Howel AW et.al** | | ☆ | ☆ | ☆ | ☆ | ☆ | ☆ |  | | *6* | High Risk of Bias |
| **Tozzoli R et.al** | | ☆ | ☆ | ☆ | ☆ | ☆ | ☆ | ☆ | | *7* | Low risk of bias |
| **Gerenova JB et.al** | | ☆ | ☆ | ☆ | ☆ |  | ☆ |  | | *5* | High Risk of Bias |
| **Checchi S et.al** | | ☆ | ☆ | ☆ | ☆ | ☆☆ | ☆ | ☆ | | *8* | Low risk of bias |
| **Lahner E et.al** | | ☆ | ☆ | ☆ | ☆ | ☆ | ☆ | ☆ | | *7* | Low risk of bias |
| **Chan JC et.al** | | ☆ | ☆ | ☆ | ☆ | ☆☆ | ☆ | ☆ | | *8* | Low risk of bias |
| **Morawiec-Szymonik E et.al** | | ☆ | ☆ | ☆ | ☆ | ☆ | ☆ |  | | *6* | High Risk of Bias |
| **Garcia-Garcia B et.al** | | ☆ | ☆ | ☆ | ☆ | ☆ | ☆ |  | | *6* | High Risk of Bias |
| **Castoro C et.al** | | ☆ | ☆ | ☆ | ☆ | ☆☆ | ☆☆ | ☆ | | *9* | Low risk of bias |
| **Alexandraki K et.al** | | ☆ | ☆ | ☆ | ☆ | ☆ | ☆ | ☆ | | *7* | Low risk of bias |
| **Yadav R et.al** | | ☆ | ☆ | ☆ | ☆ |  | ☆ |  | | *5* | High Risk of Bias |
| **Khan A et.al** | | ☆ | ☆ | ☆ | ☆ | ☆☆ | ☆ | ☆ | | *8* | Low risk of bias |
| **Kumar A et.al** | | ☆ | ☆ | ☆ | ☆ | ☆ | ☆ |  | | *6* | High risk of bias |
| **Khubchandani A et.al** | | ☆ | ☆ | ☆ | ☆ |  | ☆ |  | | *5* | High risk of bias |
| **Twito O et.a** | | ☆ | ☆ | ☆ | ☆ | ☆ | ☆ | ☆ | | *7* | Low risk of bias |
| **Choudhary O et.al** | | ☆ | ☆ | ☆ | ☆ |  | ☆ |  | | *5* | High risk of bias |
|  | **NEWCASTLE - OTTAWA QUALITY ASSESSMENT SCALE FOR COHORT STUDIES** | | | | | | | | | | |
| **STUDY** | | **SELECTION** | | | | **COMPARABILITY** | **OUTCOME** | |  |  |  |
|  | | **Representativeness of the exposed cohort** | **Selection of the non-exposed cohort** | **Ascertainment of exposure** | **Demonstration that outcome of interest was not present at start of study** | **Comparability of Cohorts on the Basis of the Design or Analysis Maximum : ☆☆** | **Assessment of outcome** | **Was follow-up long enough for outcomes to occur** | **Adequacy of follow up of cohorts** | **SCORE** | **Evidence quality** |
| **Morel S et.al** | | ☆ | ☆ | ☆ | ☆ | ☆ | ☆ | ☆ | ☆ | 8 | Low risk of bias |
| **Meling AE et.al** | | ☆ | ☆ | ☆ | ☆ | ☆ | ☆ | ☆ | ☆ | 8 | Low risk of bias |

|  | |  | | **NEWCASTLE - OTTAWA QUALITY ASSESSMENT SCALE FOR CASE-CONTROL STUDIES** | | | | | | | | | |
| --- | --- | --- | --- | --- | --- | --- | --- | --- | --- | --- | --- | --- | --- |
| **STUDY** | | **SELECTION** | | | | **COMPARABILITY** | | **EXPOSURE** | |  |  |  |  |
|  | | **Is the case definition adequate?** | | **Representativeness of the cases** | **Selection of Controls** | **Definition of Controls** | **Comparability of cases and controls on the basis of the design or analysis (Maximum : ☆☆ )** |  | **Same method of ascertainment for cases and controls** | **Non-Response rate** | **Ascertainment of exposure** | **SCORE** | **Evidence quality** |
| **Venerito M et.al** | | ☆ | | ☆ | ☆ | ☆ | ☆ | | ☆ | ☆ | ☆ | 8 | Low risk of bias |
| **Wang YP et.al** | | ☆ | | ☆ | ☆ | ☆ | ☆ | | ☆ | ☆ | ☆ | 8 | Low risk of bias |
| **Mehmet E et.al** | |  | | ☆ | ☆ |  | ☆ | | ☆ |  | ☆ | 5 | High risk of bias |
| **Adnan A et.al** | |  | | ☆ | ☆ | ☆ | ☆ | | ☆ |  | ☆ | 6 | High risk of bias |
| **Koç Şanver et.al** | | ☆ | | ☆ | ☆ | ☆ | ☆ | | ☆ |  | ☆ | 7 | Low risk of bias |
| **Nalbant A et.al** | |  | | ☆ | ☆ | ☆ | ☆ | | ☆ |  | ☆ | 6 | High risk of bias |
| **Caplan R et.al** | | ☆ | | ☆ | ☆ | ☆ | ☆ | | ☆ |  | ☆ | 7 | Low risk of bias |
| **Colleran K et.al** | | ☆ | | ☆ | ☆ | ☆ | ☆ | | ☆ |  | ☆ | 7 | Low risk of bias |
| **Alperin J et.al** | |  | | ☆ | ☆ | ☆ | ☆ | | ☆ |  | ☆ | 6 | High risk of bias |
| **Ranjan A et.al** | | ☆ | | ☆ | ☆ | ☆ | ☆ | | ☆ | ☆ | ☆ | 8 | Low risk of bias |
| **Nitu C et.al** | | ☆ | | ☆ | ☆ | ☆ | ☆ | | ☆ |  | ☆ | 7 | Low risk of bias |
| **Tripathi P et.al** | |  | | ☆ | ☆ | ☆ | ☆ | | ☆ |  | ☆ | 6 | High risk of bias |
| **Onat A et.al** | |  | | ☆ | ☆ |  | ☆ | | ☆ |  | ☆ | 5 | High risk of bias |
| **Berker D et.al** | |  | | ☆ | ☆ | ☆ | ☆ | | ☆ |  | ☆ | 6 | High risk of bias |
| **Photam S et.al** | | ☆ | | ☆ | ☆ | ☆ | ☆ | | ☆ |  | ☆ | 7 | Low risk of bias |
| **Çakal B et.al** | | ☆ | | ☆ | ☆ | ☆ | ☆ | | ☆ | ☆ | ☆ | 8 | Low risk of bias |
| **Nedrebo B et.al** | |  | | ☆ | ☆ | ☆ | ☆ | | ☆ |  | ☆ | 6 | High risk of bias |
| **Diekman MJ et.al** | | ☆ | | ☆ | ☆ | ☆ | ☆ | | ☆ |  | ☆ | 7 | Low risk of bias |

**Figure S1. Analysis of subgroups according to continents between patients with hypothyroidism vs healthy patients**

**
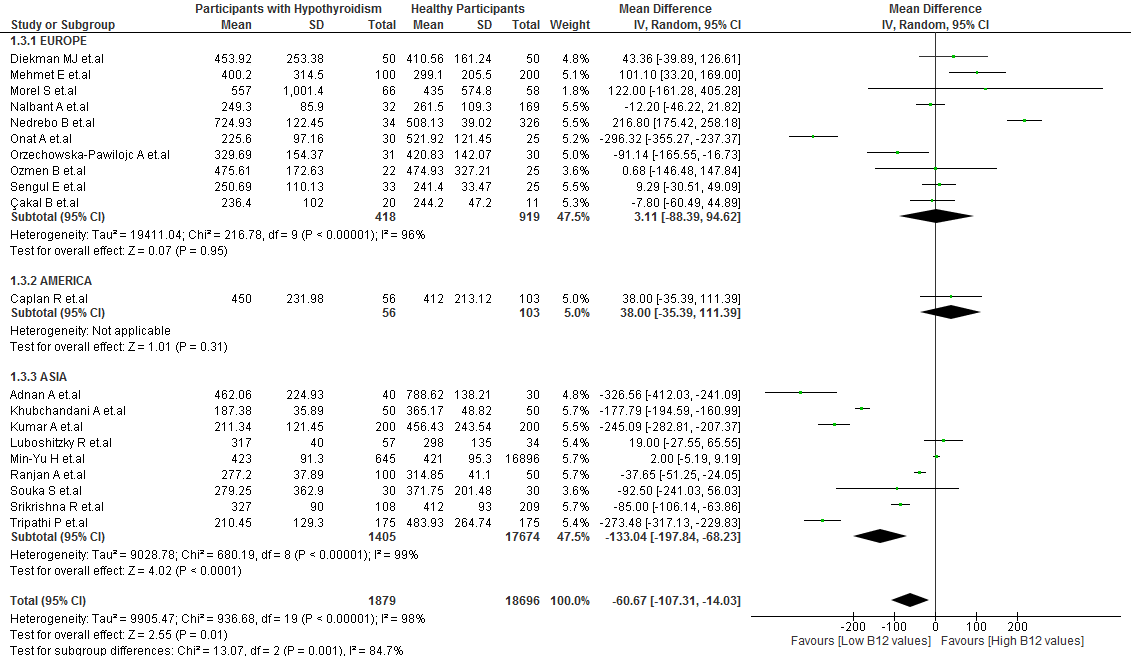
**

**Figure S2. Sensitivity analysis according to the risk of bias between patients with hypothyroidism vs healthy patients**

**
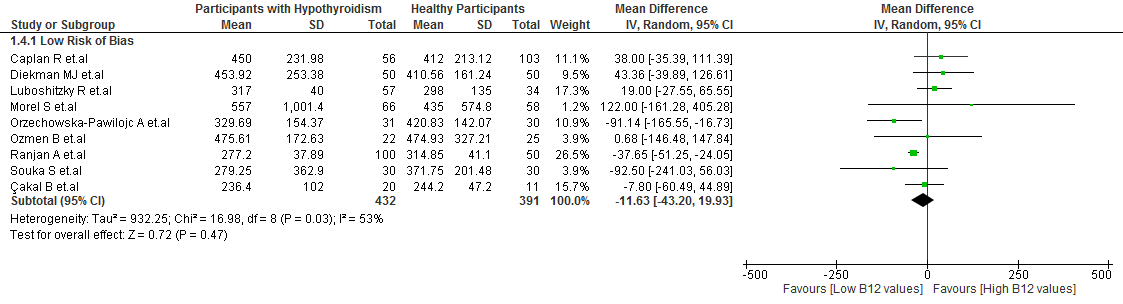
**

**Figure S3. Analysis of subgroups according to continents between patients with hyperthyroidism vs healthy patients**

**
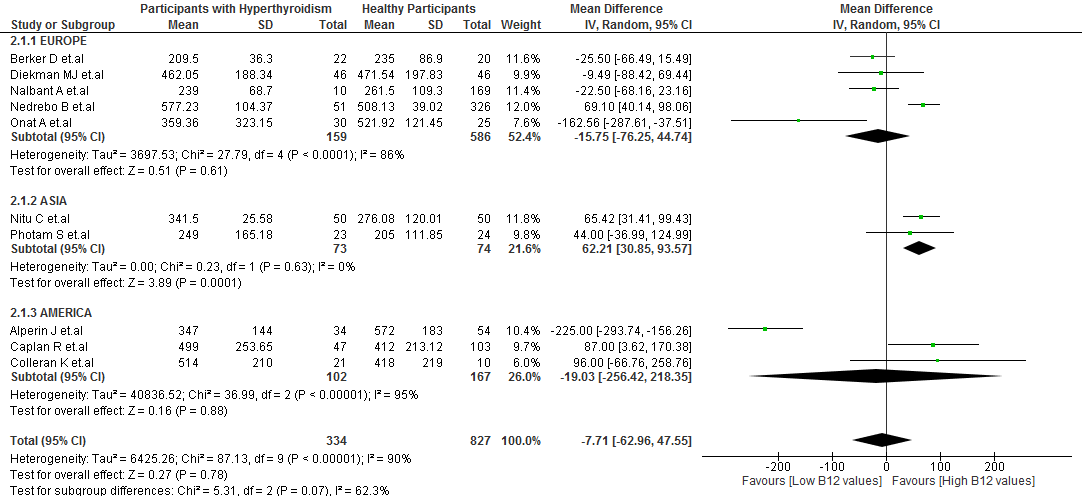
**

**Figure S4. Sensitivity analysis according to the risk of bias between patients with hyperthyroidism vs healthy patients**

**
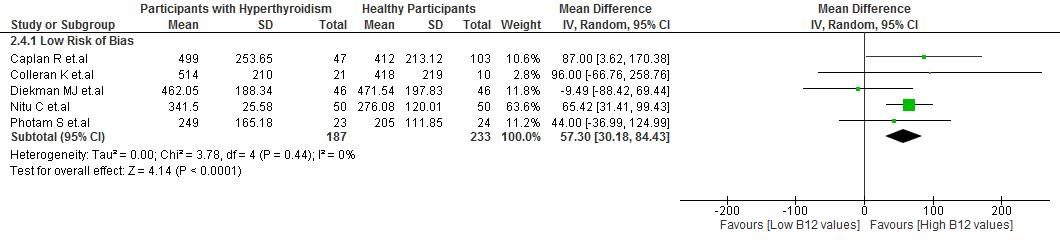
**

**Figure S5. Analysis of subgroups according to continents between patients with AITD vs healthy patients**

**
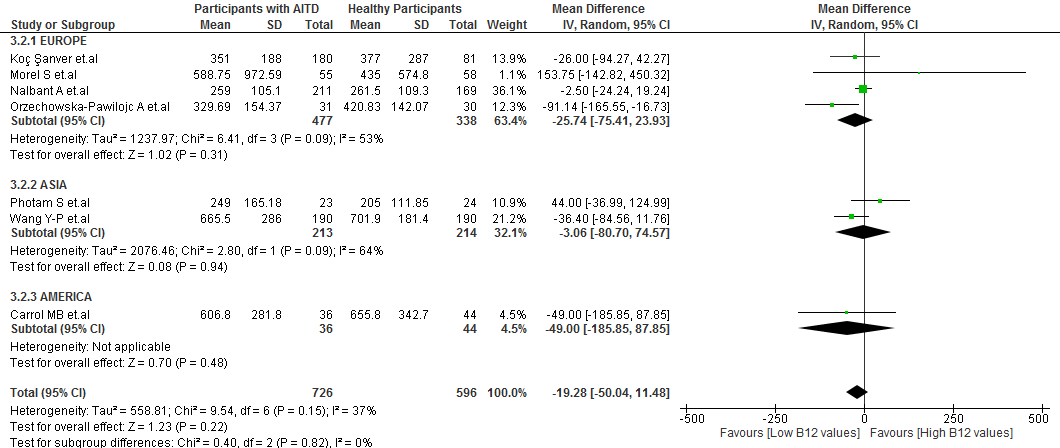
**

**Figure S6. Sensitivity analysis according to the risk of bias between patients with AITD vs healthy patients**

**
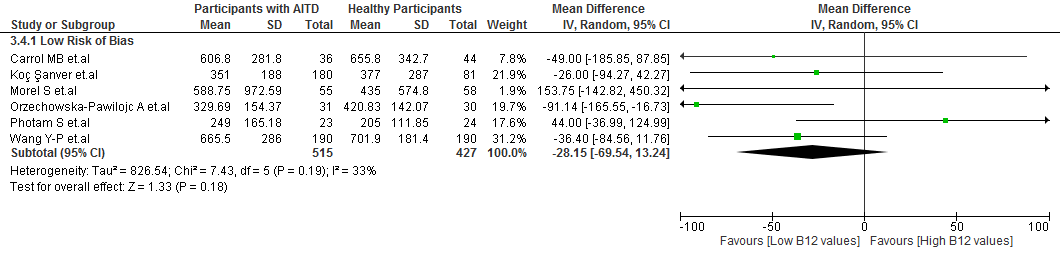
**

**Figure S7. Analysis of subgroups according to continents between patients with SH vs healthy patients**

**
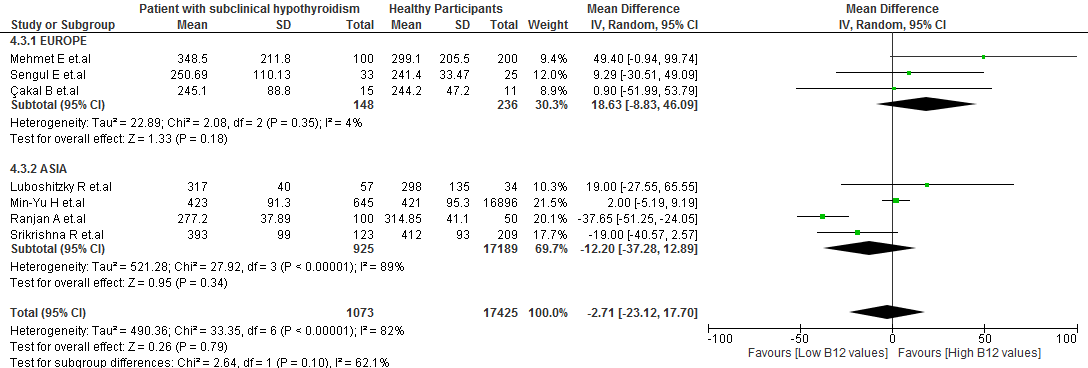
**

**Figure S8. Sensitivity analysis according to the risk of bias between patients with SH vs healthy patients**

**
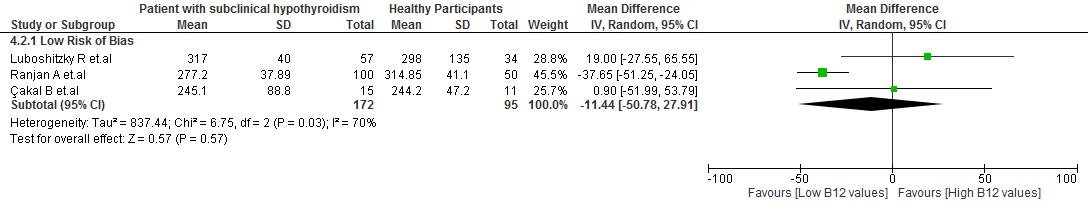
**

**Figure S9. Frequency of vitamin B12 deficiency in hypothyroidism**

**Figure S10. Sensitivity analysis of the frequency of vitamin b12 deficiency in hypothyroidism**

**
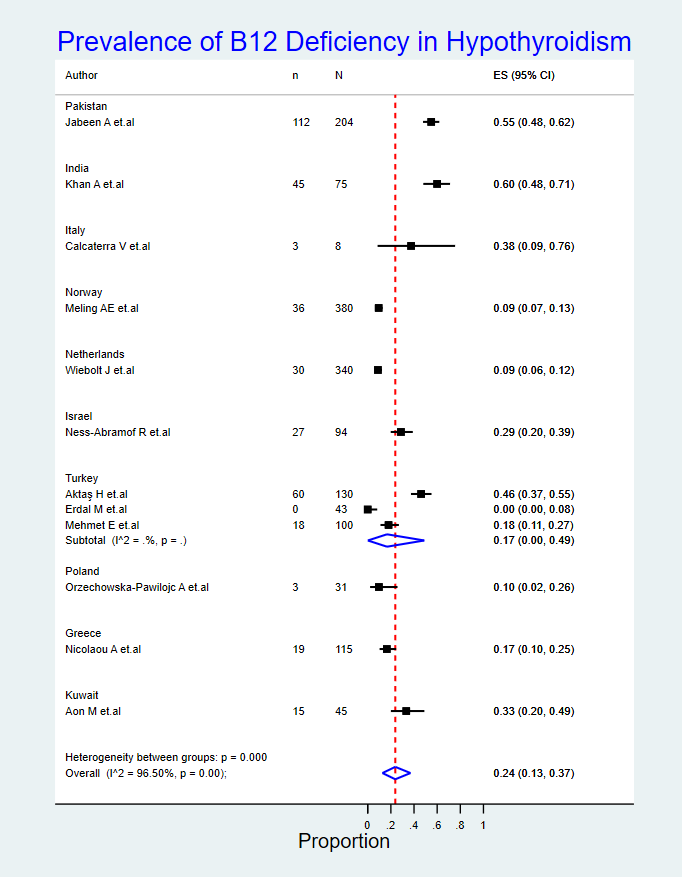
**

**Figure S11. Frequency of vitamin B12 deficiency in hyperthyroidism**


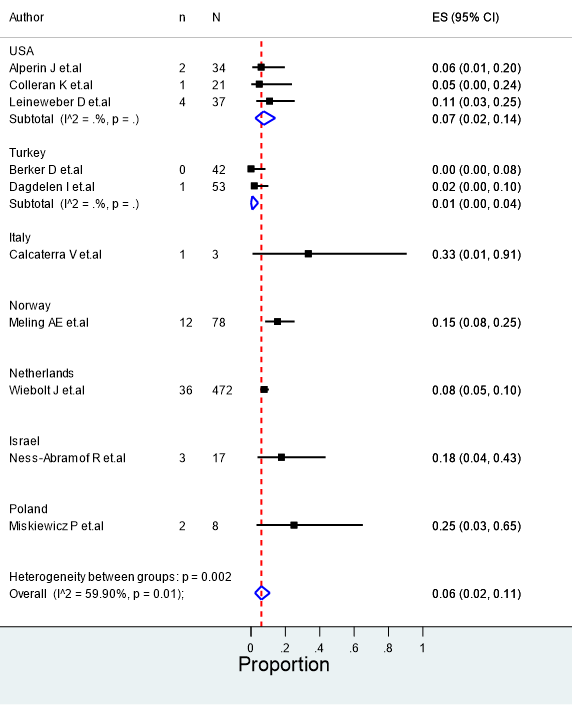


**Figure S12. Sensitivity analysis of the frequency of vitamin b12 deficiency in hyperthyroidism**

**
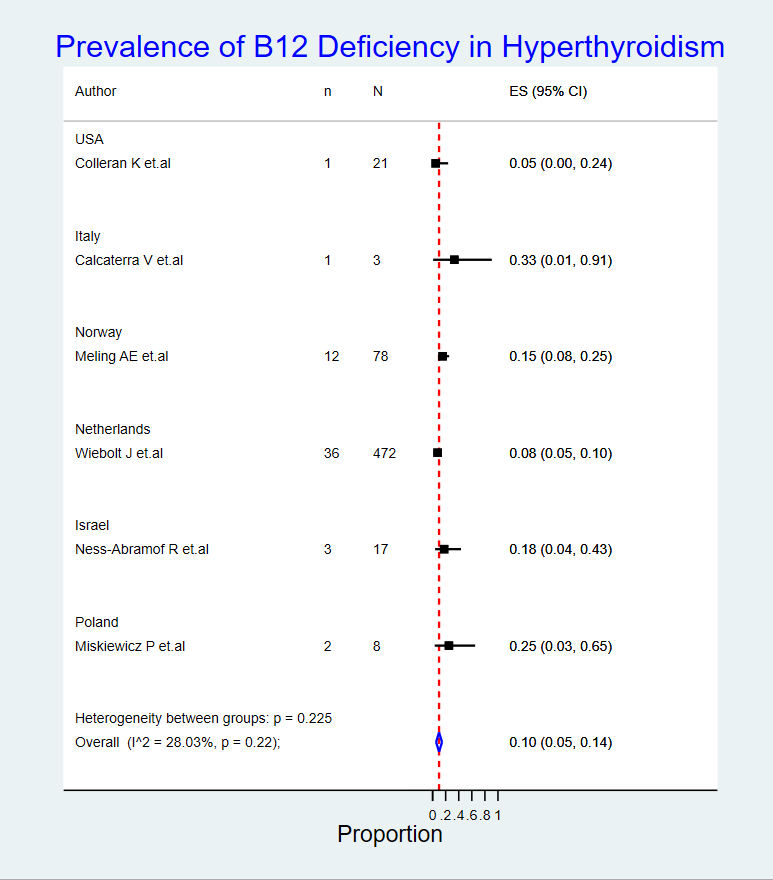
**

**Figure S13. Frequency of vitamin B12 deficiency in AITD**

**Figure S14. Sensitivity analysis of the frequency of vitamin b12 deficiency in AITD**

**
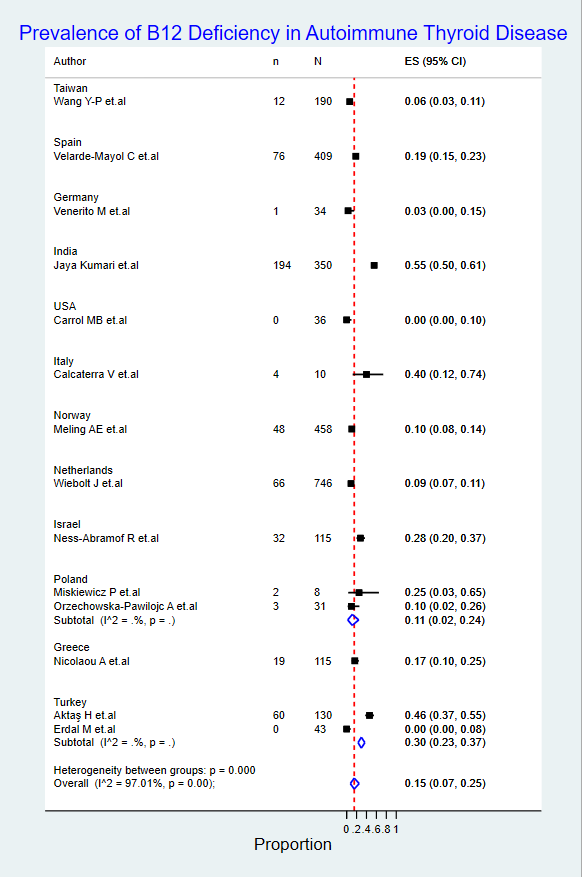
**

**Figure S15. Frequency of vitamin B12 deficiency in SH**


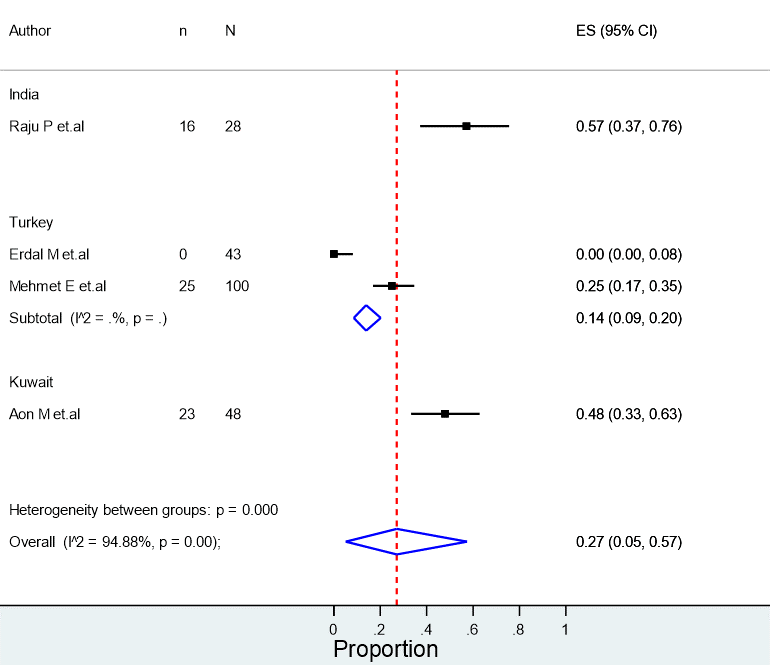


**Figure S16. Sensitivity analysis of the frequency of the presence of APCA in AITD**

**
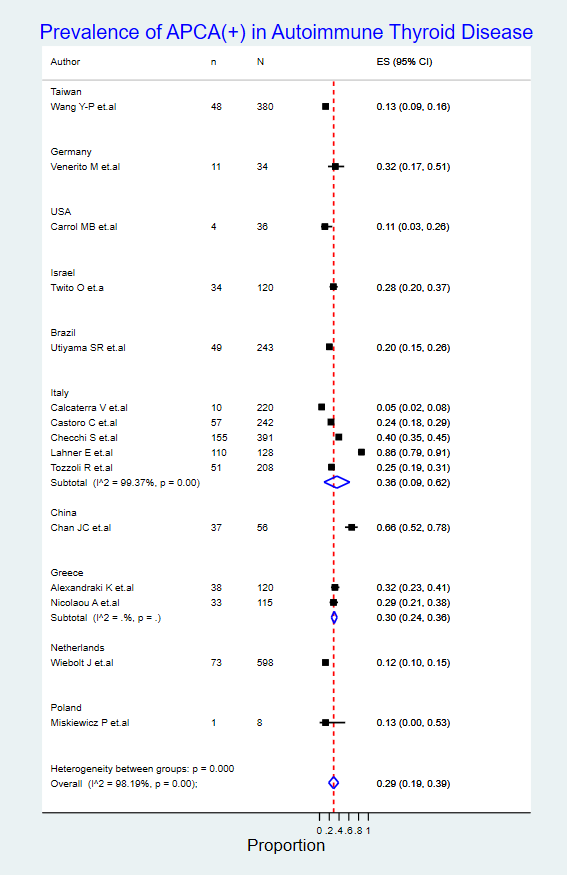
**

**Figure S17. Funnel Plot of the studies that evaluated vitamin B12 levels in hypothyroidism**

**
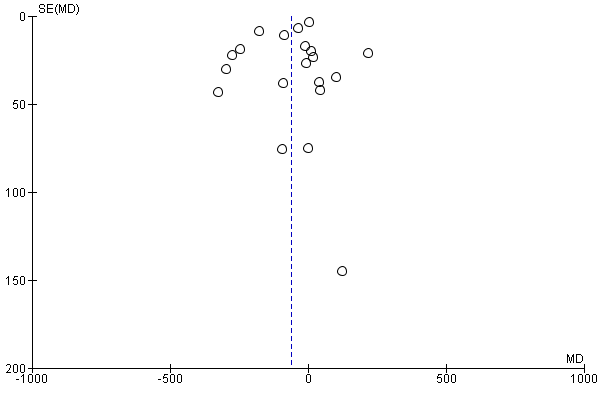
**

**Figure S18. Funnel Plot of the studies that evaluated vitamin B12 levels in hyperthyroidism**

**
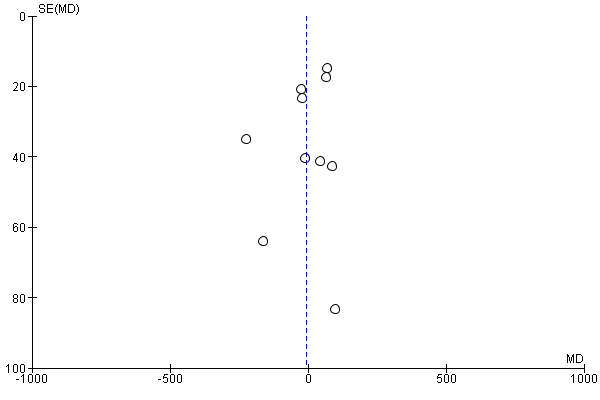
**

**Figure S19. Funnel Plot of the studies that evaluated vitamin B12 levels in AITD**

**
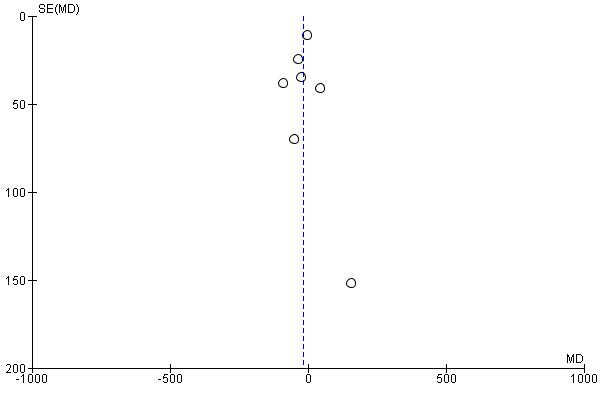
**

**Figure S20. Funnel Plot of the studies that evaluated vitamin B12 levels in SH**

**
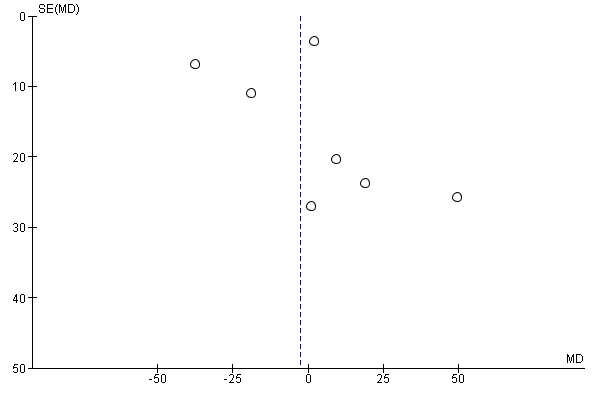
**
